# Shaping the Glycan Landscape: Hidden Relationships between Linkage and Ring Distortions Induced by Carbohydrate-Active Enzymes

**DOI:** 10.1021/jacs.5c11504

**Published:** 2025-10-09

**Authors:** Isabell Louise Grothaus, Paul Spellerberg, Carme Rovira, Lucio Colombi Ciacchi

**Affiliations:** † Hybrid Materials Interfaces Group, Bremen Center for Computational Materials Science and MAPEX Center for Materials and Processes, 9168 University of Bremen, 28359 Bremen, Germany; ‡ Malopolska Centre of Biotechnology, 117230 Jagiellonian University, 31-007 Krakow, Poland; § Department of Theoretical Biophysics, 16724 Max Planck Institute for Biophysics, 60438 Frankfurt, Germany; ∥ Faculty for Biology and Chemistry, University of Bremen, 28359 Bremen, Germany; ⊥ Departament de Química Inorgànica i Orgànica & IQTCUB, Universitat de Barcelona, Barcelona 08028, Spain; # Institució Catalana de Recerca i Estudis Avançats (ICREA), Barcelona 08020, Spain

## Abstract

Carbohydrate-active enzymes (CAZymes) catalyze glycan remodeling by forming and cleaving glycosidic bonds. An often-observed aspect of their catalytic mechanisms, particularly in glycosidases, is monosaccharide ring distortion that brings the substrate from a stable solution conformation to a reactive state. To what extent this distortion is promoted by steric constraints in the enzyme’s binding pocket or by more elusive dynamical effects associated with the glycan conformational flexibility is still a matter of debate. In our work, we quantify the conformational phase-space changes experienced by glycans upon CAZyme binding by means of enhanced-sampling molecular dynamics simulations. Our results reveal a novel correlation between torsional degrees of freedom along the glycosidic bonds and the pucker degrees of freedom within the mannose ring at the −1 subsite of glycan M5G0 upon binding to the Golgi α-mannosidase II enzyme. Key factors driving this torsional phase-space reshaping and the associated transition from solution ^4^ *C*_1_ to ^O^ *S*_2_/*B*_2,5_ reactive pucker states include tight interactions with a protonated aspartic acid and a Zn^2+^ ion in the catalytic site. Comparative studies with ER α-mannosidase I show a different mechanism, where torsional conformations and ring distortion of the M9 glycan substrate are not correlated. By validating against previous computational and experimental studies, we theoretically predict the influence of amino acid mutations and altered glycan compositions on the conformational transition mechanisms. Our findings provide new insights into CAZyme specificity and effectiveness, laying the groundwork for the design of selective inhibitors targeting glycosylation-related diseases.

## Graphical abstract

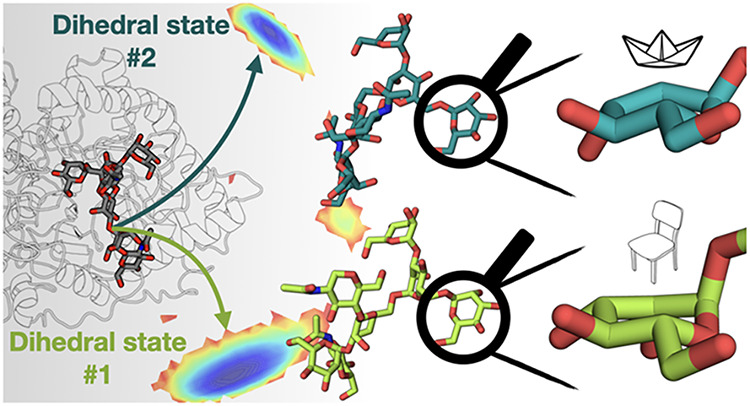

## Introduction

Carbohydrate-active enzymes (CAZymes) account for 1 to 3% of most genomes. They are responsible for the modification of the cell glycome through glycosidic bond breakage and formation, particularly by glycoside hydrolases (GH) and glycosyltransferases (GT), respectively.^1^ The vast diversity and complexity of glycan substrates require a wide portfolio of protein sequences, classified into 189 GH and 137 GT families.^2^ ^,^ [Fn fn1] Glycans are composed of sugar monomers (monosaccharides) assembled into linear or branched structures through the formation of glycosidic linkages. Owing to the diversity of monosaccharides, linkage positions, orientations, and degrees of branching, glycans inherit a much larger chemical and structural variety compared to nucleic acids and proteins. Their behavior is characterized by a high flexibility, which manifests itself in rapid transitions between individual conformers (Figure 1 A,B).^3^ These can be differentiated by the dihedral (torsional) angles φ, ψ, and ω along the glycosidic bonds, and by the Cremer–Pople parameters ϕ and θ determining the puckering of the monosaccharide rings (Figure 1 C,D).^4^ The probability distributions of these dihedral and puckering degrees of freedom adopted by a glycan during its dynamics define its conformational phase space, which captures and quantifies the occurrence of its three-dimensional structural conformers and is associated with its biological functions.

**Figure 1:**
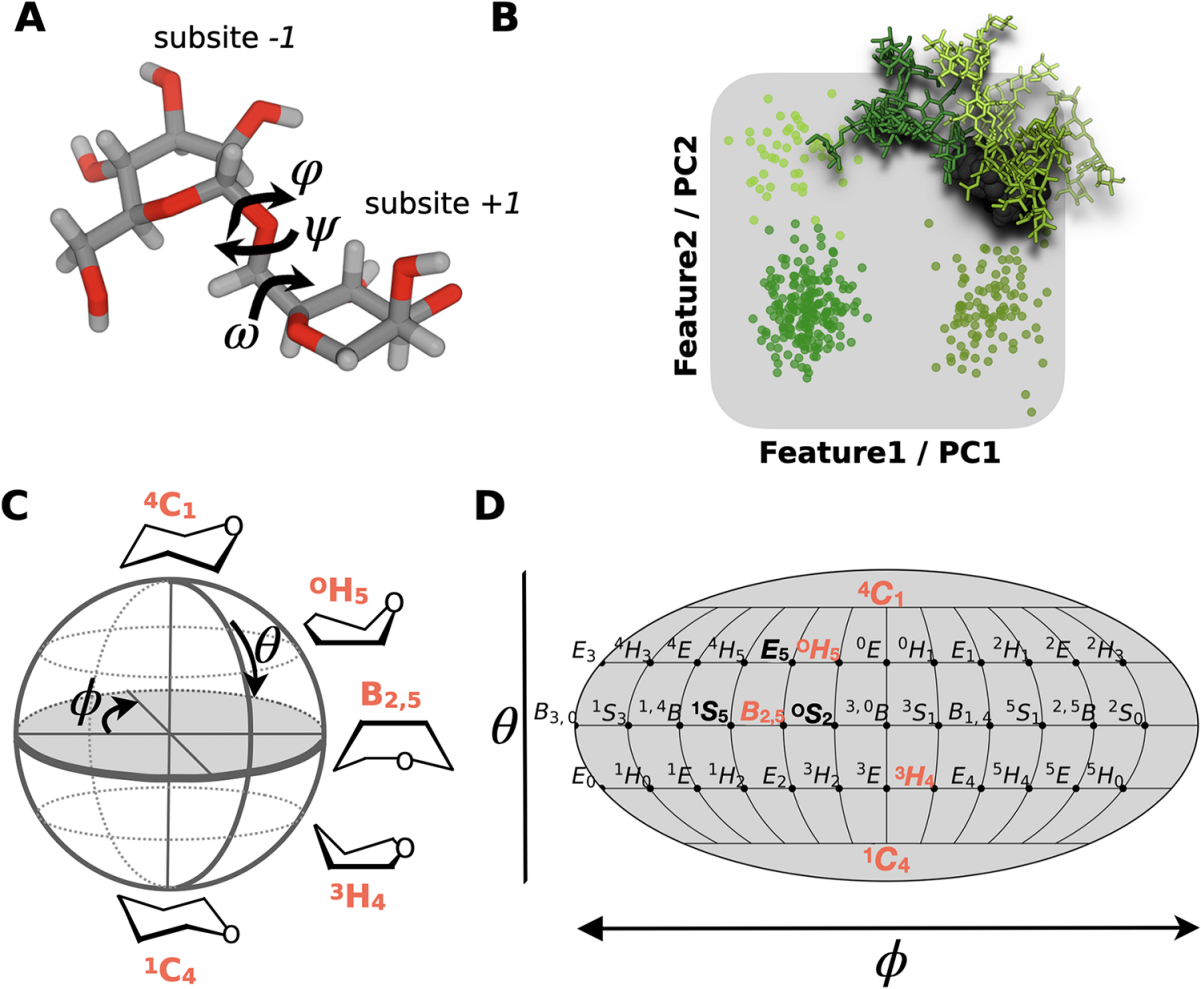
Representation of the glycan’s conformational phase space. (A) Glycans are flexible molecules, adopting various distinct conformers that are characterized by the dihedral angles φ, ψ, and ω along the glycosidic bonds connecting the individual monosaccharides e.g., mannoses located at subsite −1 and +1 in a CAZyme binding site. The atomistic structure is represented in licorice style with oxygen atoms in red, hydrogens in white, and carbons in gray. (B) The conformational phase space can be best represented in low dimensions employing features as axes that can be identified by, e.g., principal component analysis. Points in this latent space represent individual structures that are colored by their respective conformer. Atomistic structures colored in green represent three different conformers of the glycan M5G0. (C) An additional degree of freedom is the flexibility of the pyranose ring, whose distortion is quantified by the Cremer–Pople pucker coordinates θ and ϕ, and (D) plotted using a Mollweide projection, a pseudocylindrical map with equal area, meaning that areas, densities, and thus free energy values are preserved. Labels represent individual ring shapes.^4^

CAZymes evolved a remarkable binding specificity for different glycans. In the particular case of GH enzymes, the glycan substrates very often undergo a ring distortion at the monosaccharide bound at subsite *–*1 with respect to the bond cleavage point during catalysis (Figure 2 A).^5–8^ This puckering is necessary to achieve an axial orientation of the glycosidic bond, facilitating the S_N_2 reaction, irrespective of whether the hydrolytic mechanism is inverting or retaining.^5^ Especially in the case of mannosidases, the sugar ring also adopts a conformation that avoids the steric interaction between 2-OH (axial in ^4^ *C*_1_ mannose) and the catalytic nucleophile. The precise itinerary from the reactant state (also known as the Michaelis complex) to the transition and the product states along the catalytic reaction might vary for different substrates and GH families. However, ring distortions away from the most stable ring shapes ^4^ *C*_1_ and ^1^ *C*_4_ adopted in solution are likely to be observed along most reactions.

**Figure 2:**
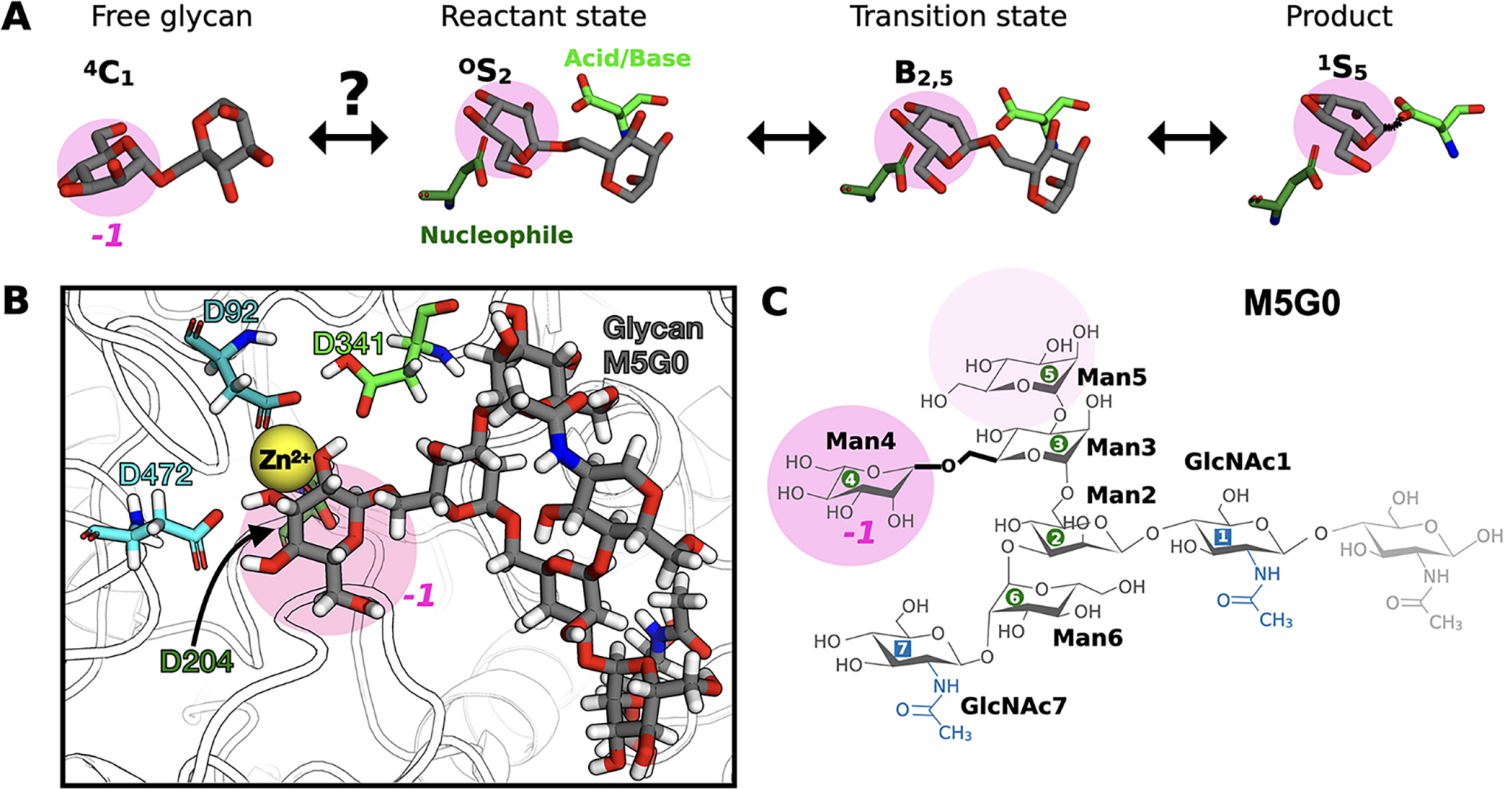
Catalytic details of Golgi α-mannosidase II. (A) The catalytic itinerary of the glycosylation step, depicting the transition from the solution equilibrium ^4^ *C*_1_ chair to the product in the ^1^ *S*_5_ skew-boat. The monosaccharide at subsite *–*1 is the residue that must be cleaved off. (B) The binding configuration of glycan M5G0 within the catalytic site, highlighting the Zn^2+^ ion and key amino acid residues (D92, D204, D341, and D472). Atoms are displayed in a licorice style: the glycan has gray carbon atoms, while amino acid carbons are color-coded by the residue type. Oxygen atoms are red, nitrogen atoms are blue, and hydrogen atoms are white. (C) Atomistic structure and nomenclature of M5G0 with labeling of linkages according to the neighboring residue numbers. The Man3-Man4 linkage is highlighted in bold and is to be cleaved by MII. In all panels, the to-be-cleaved Man4, positioned at the catalytic subsite *–*1, is emphasized with a transparent pink circle.

Despite the elucidation of numerous catalytic itineraries for CAZymes at the mechanistic level, a fundamental question remains: what drives the ring distortion, leading to a preactivated glycan substrate? Current debate centers on whether such distortion is imposed by catalytic-site residues following substrate binding, or whether it arises during the binding process as the glycan approaches and engages the pocket.^5,8^ We extend this question to consider the potential influence of the global glycan conformation since binding-induced deviations from the equilibrium could sterically favor otherwise inaccessible pucker states. Knowledge of the factors governing the conformation of the monosaccharide at subsite *–*1 is relevant for (i) a better understanding of CAZymes’ reaction mechanisms, (ii) a prediction of substrate specificity, and (iii) the design of inhibitors for therapeutic enzyme inhibitors. The latter are crucial in a wide field of applications such as antiviral medicines, treatment of genetic disorders, mitigation of diabetes, or cancer therapy.^9,10^

In this work, we investigate the origins of ring distortion for glycan substrates of α-mannosidase I (MI) and α-mannosidase II (MII). Mannosidases make up almost 10% of all GH families, and are crucial for the correct processing of protein *N*-glycosylation.^11^ Especially, MII is of great biological and medical relevance, enabling remodeling of high-mannose types to complex *N*-glycans. The enzyme is known to be overexpressed in cancer diseases of colon, skin and breast, resulting in altered glycoforms on cell membrane glycoproteins, which could be correlated with metastasis growth and disease progression.^12–14^ Indeed, inhibition of MII minimized the formation of complex *N*-glycans and was associated with reduced tumor growth and metastasis.^15^

MII catalyzes the hydrolysis of its substrate M5G0 by cleaving the α1–6 linkage between the mannose (Man) rings Man3 and Man4 (Figure 2 B,C). Studies based on X-ray diffraction and quantum mechanics/molecular mechanics (QM/MM) calculations have established that, starting from its equilibrium chair structure in solution, Man4 transitions through various boat states in the enzyme’s binding pocket during the enzymatic cleavage. The shapes so far identified along the pucker itinerary are C14⁢(solution)→S2/B2,5O→[B2,5]++→S51 .^16,17^ The *B*_2,5_-like conformation is a prerequisite for hydrolysis, because the axial orientation of the 2-hydroxyl group in a ^4^ *C*_1_ chair is unfavorable for the required nucleophilic substitution reaction at the anomeric position.^11^ Catalysis is assisted by a Zn^2+^ ion in the catalytic site, which coordinates the hydroxyl groups of Man4 and induces charge redistributions among the ring atoms along the catalytic itinerary (Figure 2 B).^16,18^ Analogously, the inverting α-mannosidase I (MI) catalyzes the hydrolysis of its substrate M9 by cleaving a terminal mannose transitioning from a ^4^ *C*_1_ chair in solution first to a ^1^ *C*_4_ chair and eventually to a ^3^ *H*_4_ half-chair.^19^ In this case, the reaction is assisted by a Ca^2+^ ion in the enzyme’s binding pocket.^20^

Current experimental insights into the pucker itineraries followed by mannosidase substrates are limited to crystal structures of Michaelis complexes or glycosyl-enzyme intermediates stabilized in mutant crystal structures. Because such experiments do not capture the global conformational phase space of glycan substrates, the dynamic processes leading to the distorted ring shape of the initial reactant state remain unexplored.^5,21^ Computational studies based on all-atom molecular dynamics (MD) simulations have the potential to shed light on this issue, provided that force-field parametrizations can capture the ring distortion of the Michaelis complex, as it is the case for MII.^16^ Although QM/MM approaches are able to capture ring distortion of individual monosaccharides at CAZymes’ subsites −1, their short accessible time scale is unable to quantify the phase space of the entire glycan structure. For this reason, here we employ enhanced-sampling MD simulations that fully account for the dynamical flexibility of glycans using the previously introduced REST-RECT methodology that ensures comprehensive sampling of their conformational phase spaces.^3^ We apply this replica-exchange-based scheme to whole glycan-enzyme complexes by utilizing a large amount of computational power, achieving tens of μs of sampling trajectories. This approach enables quantification of the phase-space reshaping of M5G0 and M9 upon binding to the catalytic sites of MII and MI, and reveals key factors underlying the characteristic ring distortions of their reactant states.

## Methods

### Setup of Simulation Systems

All-atom MD simulations were performed for the native or mutated CAZymes MII (PDB 3CZN) and MI (PDB 5KIJ) with their cocrystallized natural substrates M5G0 and M9. Initial structures and force field parameters were generated via CHARMM-GUI,^22,23^ setting the pH to 7.0 and preserving the glycan structures and components. This means that in both cases the first GlcNAc residue at the nonreducing end, which was not resolved in the crystal structures, is not included in the models. For MII, all amino acids 31–1044 were included in the model. The residue D341 was protonated, as known for the active enzyme, and the mutation D204A, used to stabilize the crystal structure, was reversed to deprotonated aspartic acid. In order to construct the modified substrate M5 in the binding site of MII, GlcNAc7 was deleted from the M5G0 structure and replaced by an OH group. For MI, all amino acids 245–696 were included in the model. The La^3+^ ion used experimentally for resolving the crystal structure from X-ray data was converted to a Ca^2+^ ion, as in the natural enzyme. The atomic coordinates of the water molecules bound to the ion were preserved because the ion hydration shell is crucial for its correct positioning in the catalytic site. Residues E602, E663, E689, D255, and H385 of MI were protonated, and a disulfide bond was introduced between residues 527 and 556. All systems were solvated using a 10 Å thick water layer in either cubic or dodecahedron boxes and neutralized using K^+^ or Cl^–^ ions, as required. Simulation setups of free glycans in solution were also constructed via CHARMM-GUI’s Glycan Modeler, based on averaged structures from the Glycan Fragment Database^22,24–26^ and solvated and neutralized as described above.

MD simulations were performed with GROMACS 2022,^27^ patched with the PLUMED package version 2.8.^28^ Force fields were selected after REST-RECT test simulations (see simulation details below) of M5G0+MII, comparing the performance of the CHARMM36 and AMBER force field family (ff19SB^29^ for protein and ions, Glycam06j-1 ^30^ for carbohydrates) in TIP3P^31^ water to that of QM/MM calculations and experiments. Particular emphasis was put on the ring distortion behavior upon binding of Man4 to the catalytic site of MII, where Petersen et al. found a distorted ^O^ *S*_2_/*B*_2,5_ conformation for the Michaelis complex via QM/MM calculations.^16^ Only the GLYCAM06j parameter set could mimic such behavior. Instead, the free energy profile of M5G0+MII using the CHARMM36 force field displays the ^4^ *C*_1_ conformation as the most stable state, still exhibiting a high energy barrier between chair and boat conformations (Figure S1). Additionally, the α-mannose ring puckering signature simulated by the GLYCAM06j force field has previously been benchmarked against ring three-bond proton–proton vicinal coupling constants obtained from ultrahigh-field NMR spectra, attesting good agreement.^32^ Therefore, the AMBER force field family was used throughout the study for all simulated systems.

The input systems were energy-minimized using the steepest-descent algorithm with a tolerance of 1000 kJ mol^–1^ nm^–1^, restraining all heavy atoms. Consecutive equilibrations of 250 ps in an NVT ensemble with restrained heavy atoms and a minimum of 1 ns in the NPT ensemble without restraints were performed prior to the production MD runs. The simulations were conducted using the leapfrog algorithm with an integration time step of 2 fs. The LINCS algorithm^33^ was applied to constrain bonds involving hydrogen atoms. Temperature control was achieved using the velocity-rescaling method^34^ with a time constant of 0.1 ps, maintaining a reference temperature of 310.15 K. The system pressure was set to 1 bar and controlled by the Parrinello–Rahman barostat with a time constant of 5 ps and a compressibility of 4.5 × 10^–5^ bar^–1^. For nonbonded interactions, the Verlet list scheme^35^ was used, updating the neighbor list every 100 steps. Electrostatic interactions were calculated using the Particle Mesh Ewald (PME) method,^36^ employing a real-space cutoff distance of 1.2 nm.

An overview of simulated systems, the applied simulation techniques, and the cumulative simulation times can be found in Table 1.

**Table 1:** Simulation Details for Different Glycans and Glycan-Enzyme Complexes^a^

enzyme	modification	glycan	technique	time
–		M5G0		
	–	M5	REST-RECT	6 μs
		M9		
MII	–	M5G0		
	restraint D341_H_ – M5G0_O6_	M5G0		
	mutation D341A	M5G0		
	mutation D204A	M5G0		
	mutation D92A	M5G0	REST-RECT	8 μs
	mutation D472A	M5G0		
	without Zn^2+^	M5G0		
	–	M5		
	–	M5G0	Steered MD	2 × 6 ns
MI	–	M9	REST-RECT	8 μs

a Additional equilibration and control simulations are reported in the Supporting Information. “Time” represents the cumulative REST-RECT sampling time of all replicas.

### REST-RECT Simulations of Glycans (within CAZymes)

To ensure thorough phase-space sampling of glycans, especially within the catalytic site, enhanced-sampling simulations were carried out using a combination of the Hamiltonian replica exchange method with solute scaling (REST2)^37,38^ and the replica exchange with collective variable tempering (RECT) method,^39^ which is based on well-tempered metadynamics (WT-MetaD).^40^ This combined approach, referred to as REST-RECT, was successfully developed specifically for glycans in a previous work^3^ and employed here for all simulated systems. Starting structures for REST-RECT were derived from equilibrium simulations, and the enhanced sampling runs were conducted in a NPT ensemble, as previously described. The entire *N*-glycan was designated as the solute region, with its Hamiltonian scaled across α replicas using scaling factors λ_α_. These factors were applied to influence long-range electrostatics, Lennard–Jones interactions, and dihedral-angle interactions. Sixteen replicas were utilized, with λ_α_ values set to 1, 0.98, 0.95, 0.92, 0.90, 0.87, 0.84, 0.81, 0.78, 0.75, 0.72, 0.69, 0.65, 0.62, 0.58, and 0.55, corresponding to an effective temperature range from 310.15 to 570 K. A geometric progression of λ_α_ values, as often employed in similar systems, proved to be less efficient for replica exchanges. Throughout the simulations, water and ions were maintained at the base temperature. The RECT component applied simultaneous biases to the dihedral angles φ, ψ, and ω occurring in glycosidic linkages through one-dimensional WT-MetaD bias potentials in each replica α. The dihedral angles were defined as φ = O5′–C1’–O*x*–C*x*, ψ = C1’–O*x*–C*x*–C­(*x*–1), and ω = O6–C6–C5–O5, with *x* being the carbon number of the linkage at the nonreducing end. Replica-specific bias factors γ_α_ were defined as 1, 1.13, 1.27, 1.43, 1.61, 1.82, 2.05, 2.31, 2.60, 2.93, 3.30, 3.72, 4.19, 4.73, 5.32, and 6 along the replica ladder. Gaussian hills were deposited every τ_G_ = 1 ps, with a width of 0.35 rad and a height determined by *h*_α_ = (*k*_B_Δ*T*_α_/τ) × τ_G_, where *k*_B_ is the Boltzmann constant, Δ*T*_α_ = *T*_0_(γ_α_ – 1) is the boosting temperature, and τ = 4 ps is the bias evolution characteristic time. Replica exchanges were attempted every 400 steps and evaluated using the Metropolis-Hastings criterion. Each of the 16 replicas was simulated for 0.5 μs, yielding a cumulative sampling time of 8 μs.

During test simulations, we occasionally observed the diffusion of glycan substrates away from the enzyme binding sites in higher-temperature replicas, due to the increased flexibility upon temperature and bias increase, limiting the effectiveness of the REST-RECT sampling scheme. Therefore, restraints of 150 kJ/mol were applied to four distances (Table 2).

**Table 2:** Restrained Distances in the MII/Substrate and MI/Substrate Complexes

enzyme	atom1	atom2	distance [nm]
MII	Zn^2+^	NE2 of His90	0.50
	Zn^2+^	NE2 of His471	0.20
	Zn^2+^	O2 of Man4 (glycan)	0.40
	Zn^2+^	O3 of Man4 (glycan)	0.20
MI	Ca^2+^	O of Thr688	0.25
	Ca^2+^	OG1 of Thr688	0.25
	Ca^2+^	O2 of Man7 (glycan)	0.25
	Ca^2+^	O3 of Man7 (glycan)	0.25

In simulations of MII without the Zn^2+^ ion, restraints were not applied. We ensured that distance restraints did not influence the pucker propensity of glycan substrates by comparing the results to those of unrestrained simulations of M5G0 + MII and M9 + MI (Figure S2).

For simulations of M5G0 + MII at a fixed D341_H_–M5G0_O6_ distance, a harmonic restraint was applied with an equilibrium distance of 0.15 nm and a force constant of 5000 kJ mol^–1^ nm^–1^.

REST-RECT simulations of free glycans in solution required only 12 replicas to span a temperature range from 310.15 to 800 K. This was realized by a geometric progression of λ_α_ between 1 and 0.42 and γ_α_ between 1 and 14, which was already shown to ensure effective sampling of glycans.^3^ Sufficient replica exchanges were validated by analyzing replica transitions along the ladder and calculating the probability of replica exchanges and round-trip times. Additionally, convergence of glycan conformer distributions was assessed by checking the moving average of dominant glycan conformers over time, for consistency (see SI).

### Free Energy Reconstructions

We note that apart from distance restraints that prevented diffusion of the glycan from the binding site, the ground replicas were unbiased due to their associated factors λ_0_ = 1 and γ_0_ = 1. Application of the weighted histogram analysis method (WHAM) in order to reweight the ground-replica distributions under consideration of the influences from the distance restraints did not reveal any significant differences compared to directly using the unbiased ground replicas. We therefore neglected the usage of WHAM and used ground replicas directly in all subsequent analyses. Coordinates and variables were saved every 10 ps or more often, resulting in at least 50,000 data points per simulation. Free energies were calculated from the probabilities *P* according to the relation Δ*G* = –*k*_B_ *T* ln­(*P*). One-dimensional free energy profiles were calculated for the Cremer–Pople pucker coordinate θ, using block averaging in order to improve statistics, separating the data set into evenly distributed blocks (5 to 10, depending on the individual system). The average of all blocks $\mathrm{X̅}=\frac{1}{N}\sum_{j=1}^{N}X_{j}$ was calculated over *N* blocks, where *X*_*j*_ is the average calculated within each *j*th block. Error bars were calculated as standard deviations of the sampling distributions (standard error of the mean) $\mathrm{std}\left(\mathrm{X̅}\right)=\sqrt{\frac{\mathrm{var}\left(\mathrm{X̅}\right)}{N}}$ , with the variance of the sampling distributions

$$\mathrm{var}\left(\mathrm{X̅}\right)=\left(\frac{N}{N-1}\right)[\frac{1}{N}\overset{N}{\underset{j=1}{\sum }}X_{j}^{2}-{\left(\frac{1}{N}\overset{N}{\underset{j=1}{\sum }}X_{j}\right)}^{2}].$$

Two-dimensional pucker histograms along ϕ and θ with 200 × 200 bins were constructed using PLUMED and plotted using the Mollweide (also termed elliptical) projection. This pseudocylindrical map projection fullfils the equal-area condition, meaning that areas, densities, and thus free energy values are preserved in the two-dimensional (2D) representation. The reduced conformational phase space of a glycan was represented in a latent space by principal component analysis (PCA), calculated using the scikit-learn package.^41^ The sin and cos values of all dihedral angles φ, ψ, and ω of each glycan were defined as features (*n*_features_), resulting in a feature matrix **X** with shape *n*_samples_ × *n*_features_. This correctly accounts for the dihedral-angle periodicity. Whenever two **X** from separate simulations were compared to each other, e.g., unbound versus bound glycan, the corresponding data sets were concatenated prior to PCA calculation. Free energies (Δ*G*) along principal components 1 and 2 defining the low-dimensional latent-space matrix **T** were calculated by constructing two-dimensional histograms with 35 bins and converting the histogram probabilities *P* according to the free energy equation above.

### Steered MD Simulations

Steered MD simulations were performed for native MII with bound M5G0, using a snapshot from the REST-RECT simulations as the starting configuration where M5G0 adopts the #1* conformer (vide infra for the conformer labeling), in which the Man4 saccharide is in a chair conformation. A minimization and subsequent production run of 6 ns simulation in the NPT ensemble was performed with the same simulation parameters as before. The PLUMED package with its module MOVINGRESTRAINT was employed to apply harmonic restraints to certain variables in order to artificially alter the conformation of the glycan. Two different sets of steered MD simulations were performed. In the first set, restraints were applied to φ, ψ, and ω in the Man2-Man3 linkage as well as to ψ and ω in the Man3-Man4 linkage, with force constants of 500 kJ mol^–1^ nm^–1^ for all angles. Especially these dihedral angles have been chosen because they clearly distinguish between the #1* and #2* conformers. In Man2-Man3, φ was varied between 1.0 and 3.0 rad, ψ between 3.0 and −2.0 rad, and ω between 3.0 and 1.0 rad. In Man3-Man4m, ψ was varied between 1.0 and 3.0 rad, and ω was varied between 1.0 and −1.0 rad. In the second set, solely θ of Man4 was varied between 0.25 and 1.5 rad to drag the system from the ^4^ *C*_1_ chair to a distorted boat/skew-boat. Each steered-MD cycle lasted 3 ns, with 0.5 ns of equilibration, followed by 0.5 ns of ramping and 1 ns of equilibration, prior to 0.5 ns of ramping back to the original conditions and 0.5 ns of final equilibration. Variables were outputted and plotted every 10 ps.

The simulation of M5G0 restrained in its #2* conformer was enabled by the application of harmonic restraints on all linkage dihedral angles using force constants of 50 kJ mol^–1^ nm^–1^. Sampling of puckering landscapes was achieved using REST2, without any explicit biases on dihedral angles, as these were fixed. The same number of replicas and scaling parameters were used as already described for free glycans in solution.

### Correlation Analysis

The Pearson correlation coefficients were computed by using the pairwise correlation method implemented in Pandas. Pairwise correlations between dihedral angles and pucker coordinates were calculated and combined in a correlation matrix. As for the PCA analysis, all dihedral angles (φ, ψ, and ω) of each glycan were converted into their sin and cos values, in order to account for angle periodicity. For the correlations between glycan conformers and pucker coordinates, first, the conformers were defined based on the glycan dihedral angles, and individual labels were assigned by means of the GlyCONFORMER package.^3^ As conformer labels are ordinal, meaning categorical without a known distance between them, one-hot encoding was employed to convert conformer values into a binary format and subsequently compute pairwise Pearson coefficients.

### Classifying Glycan Conformations via GlyCONFORMER

The GlyCONFORMER code identifies and classifies glycan conformers from MD simulations by assigning a unique string based on the values of glycan linkage dihedral angles.^3^ Each glycosidic linkage contributes at least two dihedral angles (φ and ψ), with an additional ω angle for 1–6 and 2–6 linkages. A conformer is represented by a letter string with a length *n* equal to the number of dihedral angles in the glycan. The letters represent the radian values adopted by the dihedral angle, following the IUPAC nomenclature.^42^ For φ and ψ they correspond to the following radian intervals: *C* = (−0.52, + 0.52); *G*_+_ = (+0.52, + 1.57); *A*_+_ = (+1.57, +2.62); *T* = [+2.62, π] or (−π, −2.62); *A*_–_ = (−2.62, −1.57); *G*_–_ = (−1.57, −0.52). And for ω: gg = (−2.62, 0); gt = (0, 2.62); tg = [2.62, π] or (−π, −2.62). The string starts at the free reducing end and includes letters for φ, ψ, and ω (if present) in this order. At branching junctions (e.g., α1–6 and α1–3), a separator labeled by the C atom at the branch origin (e.g. **6**−) is added. The string proceeds along the higher C atom branch (e.g. 6) to the terminal residue before returning to follow the lower branch (e.g. 3). The assignment involves calculations of the free-energy profiles along each dihedral angle from converged (typically enhanced-sampling) MD trajectories. The local minima in these profiles are labeled according to the nomenclature above, and the same label is inherited by all angles in each free-energy basin around the minimum. A scheme describing the GlyCONFORMER string generation procedure for the model glycan A4G4S4 can be found in Figure S3. Conformer distribution plots for individual glycans were achieved by constructing histograms using the individually identified conformers as bins. For each frame in the trajectory, the value of each dihedral angle (φ, ψ, and ω) was recorded.

## Results and Discussion

### The Reactant State: How Is It Reached?

We start our investigation by following the pucker itinerary of M5G0 in various conditions, demonstrating the usefulness of the REST-RECT methodology for sampling the conformational phase space effectively (Figures S4–S11).^3^ In solution, Man4 adopts the expected low-energy ^4^ *C*_1_ chair conformation (Figure 3 A). Binding to MII substantially alters the puckering landscape, acquiring a second minimum around the *E*_5_/^O^ *H*_5_ envelope/half-chair (Figure 3 B).^43^ This is achieved by a 30 kJ/mol reduction in energy barrier for the ring-distortion along θ from ^4^ *C*_1_ toward the equatorial region, where the minimum corresponds to the ring shape that was experimentally determined for the acid–base MII mutant D341N (Figure 3 B).^17^

**Figure 3:**
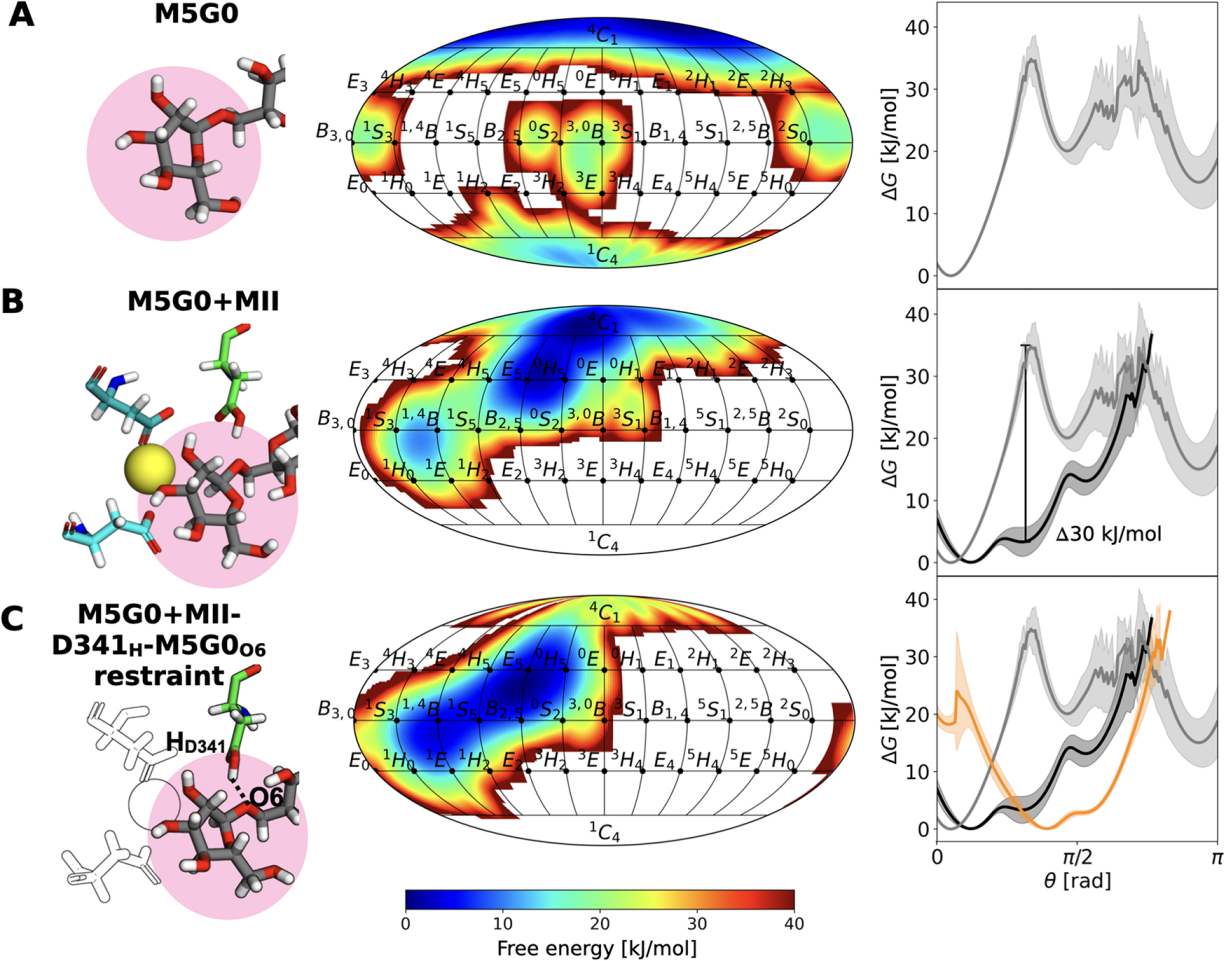
Ring puckering in different chemical environments. Ring distortion of the terminal Man4 at subsite *–*1 (highlighted in pink in the atomistic snapshot) in M5G0 monitored by the 2D Cremer–Pople representation along ϕ and θ as well as 1D along θ in different chemical environments: (A) M5G0 in aqueous solution (gray), (B) M5G0 in the catalytic site of MII (black), and (C) M5G0 in the catalytic site of MII with a distance restraint (dotted black line in the atomistic snapshot with only important residues highlighted by colors) between the O6 of M5G0 and the H atom of the carboxyl group of D341 (orange). Error bars are plotted as shaded regions, representing the standard deviation calculated from block averaging.

The reactant-state ^O^ *S*_2_/*B*_2,5_ conformation described by Petersen et al. can only be fully achieved by application of a distance restraint on D341_H_–M5G0_O6_ (Figure S12A), which leads to a broadening and shift of the global energy minimum (Figures 3 C and S12A).^16^ We think that the underestimation of the hydrogen bond distance is due to the lack of polarizability of the fixed-charge force field employed in our simulations. In fact, mimicking a polarization along the C1’–O6 bond via larger partial charges leads to a spontaneous reduction of the D341_O_–M5G0_O6_ and D341_OH_–M5G0_O6_ distances as well as a further ring distortion toward the equator in a conventional MD simulation (Figure S12B), implicating that a charge redistribution may be required to reach a ^O^ *S*_2_/*B*_2,5_ conformation (Figure S12C).

In contrast to earlier claims that the *B*_2,5_ conformation is necessary for the terminal mannose residue Man4 to fit into the catalytic site of MII,^17^ our simulations reveal that the solution ^4^ *C*_1_ chair conformation also perfectly fits into the cavity. The binding of M5G0 to MII only shifts the likelihood of Man4 puckering away from the chair conformation toward distorted shapes that activate Man4 for chemical attack. The ring distortion is, therefore, not a prerequisite for binding. It is rather an effect of confinement within the catalytic site, assisted by charge redistribution. In the next section, we examine in more detail the effect of certain modifications at the catalytic site on ring puckering.

### The Reactant State: How Is It Influenced?

The specificity of protein–glycan interactions responsible for ring distortion was investigated by REST-RECT simulations of the four mutants D341A, D204A, D92A, and D472A (Figure S13), as well as by deletion of the Zn^2+^ ion. These amino acids were selected based on the variation of their distances from Man4 depending on the ring shape (^4^ *C*_1_ or *E*_5_/^O^ *H*_5_), as observed in unbiased MD simulations (Figure S13).

We first note that our simulation approach is able to reproduce the behavior of both the D341A mutation, actually observed for D341N (PDB: 3BUP), and the D204A mutation (PDB: 3CZN) that have experimentally been shown to be catalytically inactive. D341N removes the catalytic acid/base residue from the active site that primarily serves as a crucial hydrogen donor/acceptor for the catalytic reaction but is not required for preactivation of the substrate, as a distorted *E*_5_/^O^ *H*_5_ conformation at subsite −1 persists (Figure 4 A). Mutation of the catalytic nucleophile D204A, however, results in a lack of distortion of Man4 away from its solution structure ^4^ *C*_1_. Indeed, the free energy profile along θ from the enhanced sampling simulation resembles that of a free glycan in solution (Figure 4 B). This is consistent with the crystal stucture of the D204A mutant.^17^

**Figure 4:**
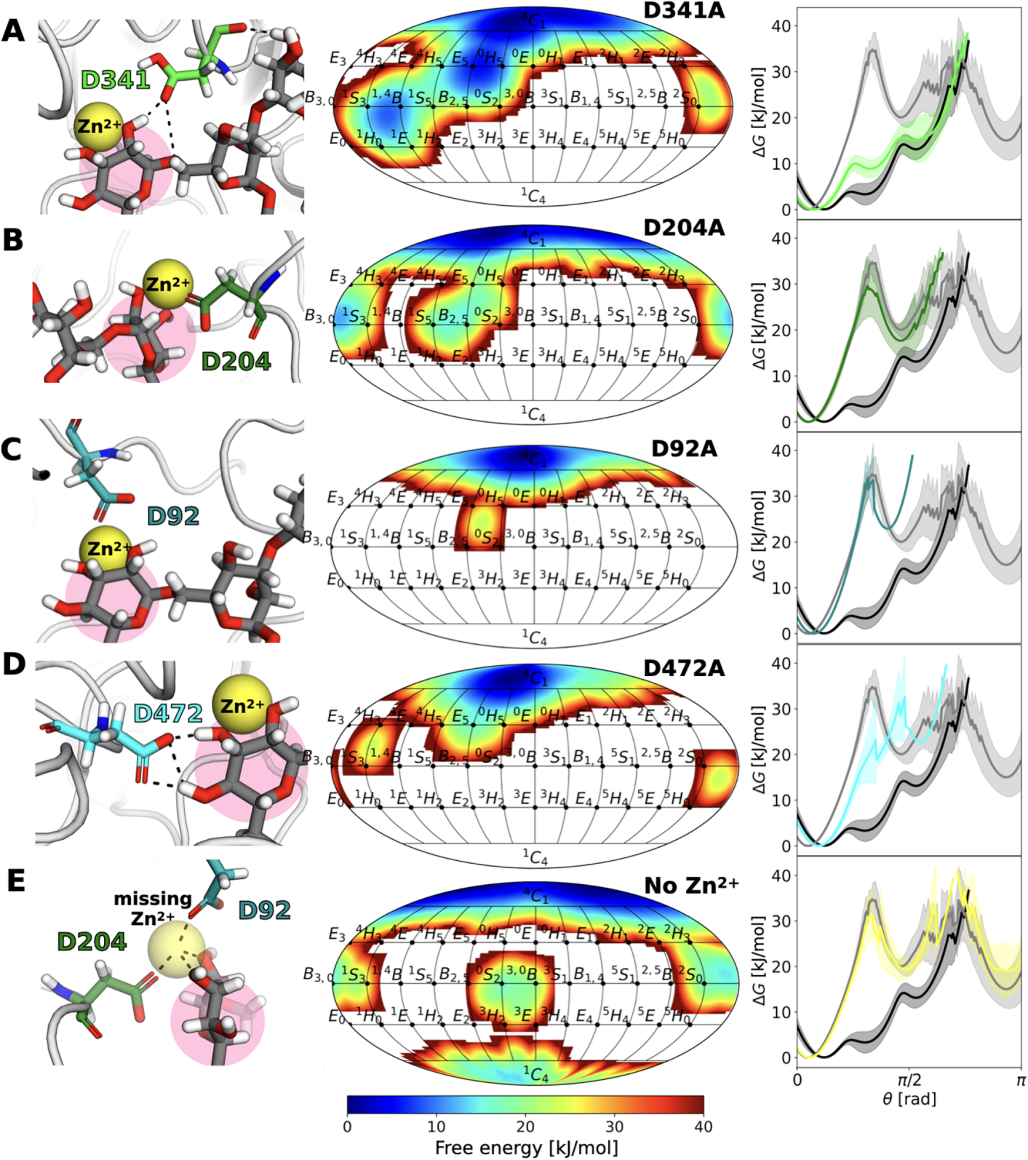
Mutants altering ring distortion. For (A) M5G0+MII-D341A, (B) M5G0 + MII-D204A, (C) M5G0 + MII-D92A, (D) M5G0 + MII-D472A, and (E) absence of the Zn^2+^ ion, the following separate panels are shown: atomistic snapshot of the catalytic site under native conditions (left), free energy surface along the Cremer–Pople puckering parameters θ and ϕ for the terminal Man4 of glycan M5G0 (highlighted in pink in the left panels) bound to mutant MII (center), and free energy profile along θ of Man4 for the unbound (gray), bound (black), and altered state (colored) (right).

After having ensured that we are able to reproduce experimental findings using the REST-RECT methodology and the GLYCAM06j force field, we address the effect of mutations D92A and D472A on ring puckering, which have not been tested experimentally so far. Both mutants are likely catalytically inactive, as ring distortion is completely compromised due to incorrect positioning of the Zn^2+^ ion for M5G0 + MII-D92A or a lack of hydrogen bonds to Man4 for M5G0 + MII-D472A (Figure 4 C/D).

Further, QM/MM calculations suggested that the Zn^2+^ ion in the catalytic site of MII helps in activating the ring distortion and stabilizing the electronic charge shifts crucial for reaching the reaction’s transition state.^16^ Removal of the Zn^2+^ ion leads to an energy profile identical to that of free M5G0 in solution (Figure 4 E). This is in line with experiments showing no activity for MII lacking Zn^2+^ in the catalytic site and underlines its direct role in ring distortion.^44^

In summary, the application of the REST-RECT approach has revealed the complete system-dependent ensemble of pucker states of M5G0 at subsite −1. Our results indicate that the ^4^ *C*_1_ chair conformation can be accommodated in the binding cavity, implying that ring distortion occurs within the catalytic site, driven by specific amino acid and ion residues rather than prior to binding.

### The Reactant State: How Do Glycan Conformations Matter?

The term conformation in the context of CAZyme’s catalytic mechanisms predominantly refers to ring puckering, whereas the global glycan shape has been largely overlooked, despite the importance of ligand flexibility in protein–ligand interactions and docking affinity. Notably, our enhanced-sampling protocol not only captures ring puckering but also resolves the distribution of global sugar conformers associated with dihedral angles φ, ψ, and ω along the glycosidic bonds. Each glycan conformer is assigned a GlyCONFORMER string, representing the adopted values of each dihedral angle. Glycan structures are categorized as different conformers when one of their dihedral angles differs in terms of occupied free energy minima (see Figures S4,S4D and S5A). As the GlyCONFORMER strings are rather lengthy, we use short notations for the glycan conformers of M5G0 (#1-#6) and M5G0+MII (#1*, #2*) (Figures S4A and S5A).

PCA-reconstructed two-dimensional free energy surfaces that represent the accessible conformational phase space reveal that M5G0 in solution can adopt multiple conformers (shortly labeled #1-#6) that differ primarily in the ω angles of the 1–6 linkages (Figures 5 A and S4). In stark contrast, M5G0 bound to MII is confined to just two dominant global conformers (labeled #1* and #2*, Figure S5), which differ significantly from the conformers observed in solution (Figure S14). This shift reflects the tight binding of almost all monosaccharide residues in M5G0 to the catalytic, holding, and anchor site of MII.^45^ A putative relationship between global glycan conformation and ring distortion of Man4 in MII becomes apparent, as the ^4^ *C*_1_ chair is preserved only for the #1* conformer, whereas in the #2* conformer Man4 experiences a transition to the half-chair ^O^ *H*_5_ (Figure 5 A inserts). In solution, all #1–#6 M5G0 conformers present a ^4^ *C*_1_ ring shape. This correlation can be quantified by the computation of the pairwise Pearson correlation coefficients between global glycan conformers (defined by their dihedral angle values) and the pucker coordinate θ of Man4 (Figure 5 B). The dihedral angle and puckering degrees of freedom are all completely uncorrelated for M5G0 in solution. In contrast, MII-bound M5G0 exhibits strong positive and negative correlations between θ and conformers #2* and #1*, respectively.

**Figure 5:**
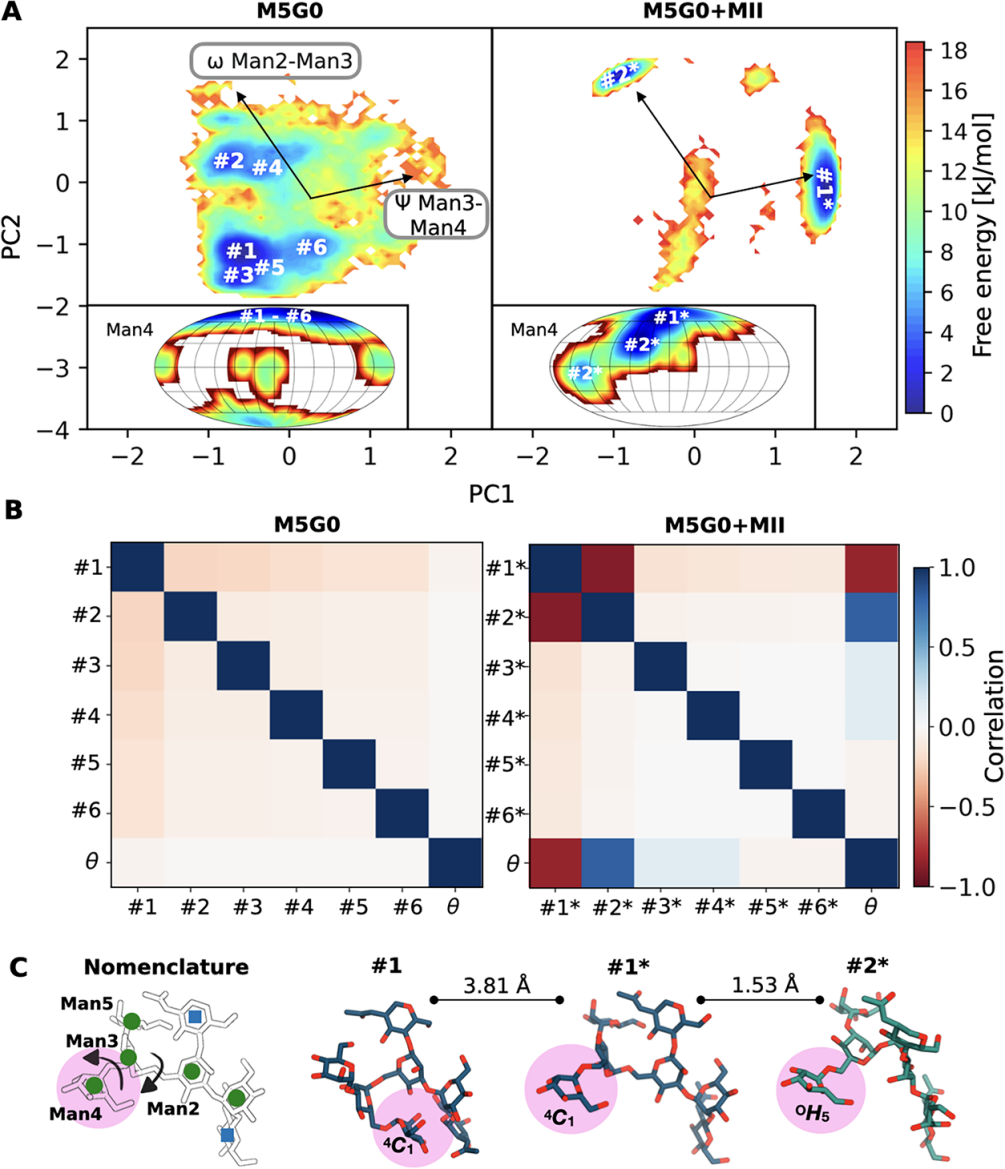
Dihedral angle-puckering correlation of M5G0 in MII. (A) Comparative free energy surfaces of the conformational phase space for M5G0 in solution and bound to MII projected along PC1 and PC2. Vectors indicate the original feature axes with the highest variance, pointing in the direction with the highest squared multiple correlation with the principal components. The inserts show the corresponding free energy surfaces along θ and ϕ for the terminal Man4, with minima labeled by the respective global glycan conformers adopted in that puckering region. (B) Correlation matrix for glycan conformers and θ of Man4 for M5G0 in solution and bound to MII, utilizing the Pearson correlation coefficient. (C) Atomistic snapshot of M5G0, highlighting the differences between conformers #1, #1*, and #2*, as indicated by arrows in the schematic structure on the left. RMSD values between conformers were calculated for the averaged structures. Conformer details in Figures S4–S5.

Further detailed analyses of correlations between dihedral angles and ring shapes are available (Figure S15), revealing specific dihedral angles, primarily within the Man2-Man3 and Man3-Man4 linkages, that strongly correlate with θ of Man4 and with each other in MII-bound M5G0 (Figure 5 C). Structural comparisons of the conformers clearly show that the shift from #1 to #1* is mainly driven by rotations around dihedral angles in the Man3-Man4 linkage (see values in Table S1), leading to a complete rearrangement of the entire glycan branch (Figure 5 C). Similarly, the transition from #1* to #2* involves a structural reorganization of the Man2-Man3 and Man3-Man4 linkages (compare with Table S1). This shift coincides with Man4 adopting the ^O^ *H*_5_ conformation.

Analysis of M5G0 conformers in mutated MII variants reveals a distinct preference for the #1* conformer, accompanied by a significant reduction in the #2* conformer (Figure S16). This observation aligns with experimental observations showing reduced enzyme activity for some of the mutants,^17,45^ emphasizing the correlation between linkage and ring distortions. We further restricted the three-dimensional (3D) structure of a free M5G0 glycan in solution to the #2* conformation in order to test if solely the dihedral angles defining the glycan conformation may be responsible for the shift in pucker propensity. However, in this case, the Man4 remains in the ^4^ *C*_1_ chair, suggesting that binding to the catalytic site is necessary to both restrict the glycan conformers and activate the ring distortion (Figure S17).

To further investigate the causality underlying the formation of the reactant state, we conducted two sets of steered MD simulations: a first one by alternating between conformers #1* and #2* using restraints on selected dihedral angles of M5G0 (Figure 6 A), and a second one by varying θ values of Man4 between a ^4^ *C*_1_ chair and boat/skew-boat ring structure (Figure 6 B). Our results reveal that upon inducing a transition to the #2* conformer, Man4 concurrently shifts to the half-chair ^O^ *H*_5_. Doing the opposite, forcing a Man4 ring distortion toward the equator, has a smaller effect on the ψ and ω dihedral of Man3-Man4 (Figure 6 B). In both cases, the D341_OH_–M5G0_O6_ distance follows the behavior of the pucker distortion with distances of 0.4 nm for ^4^ *C*_1_ and 0.3 nm for ^O^ *H*_5_ (Figure 6 C).

**Figure 6:**
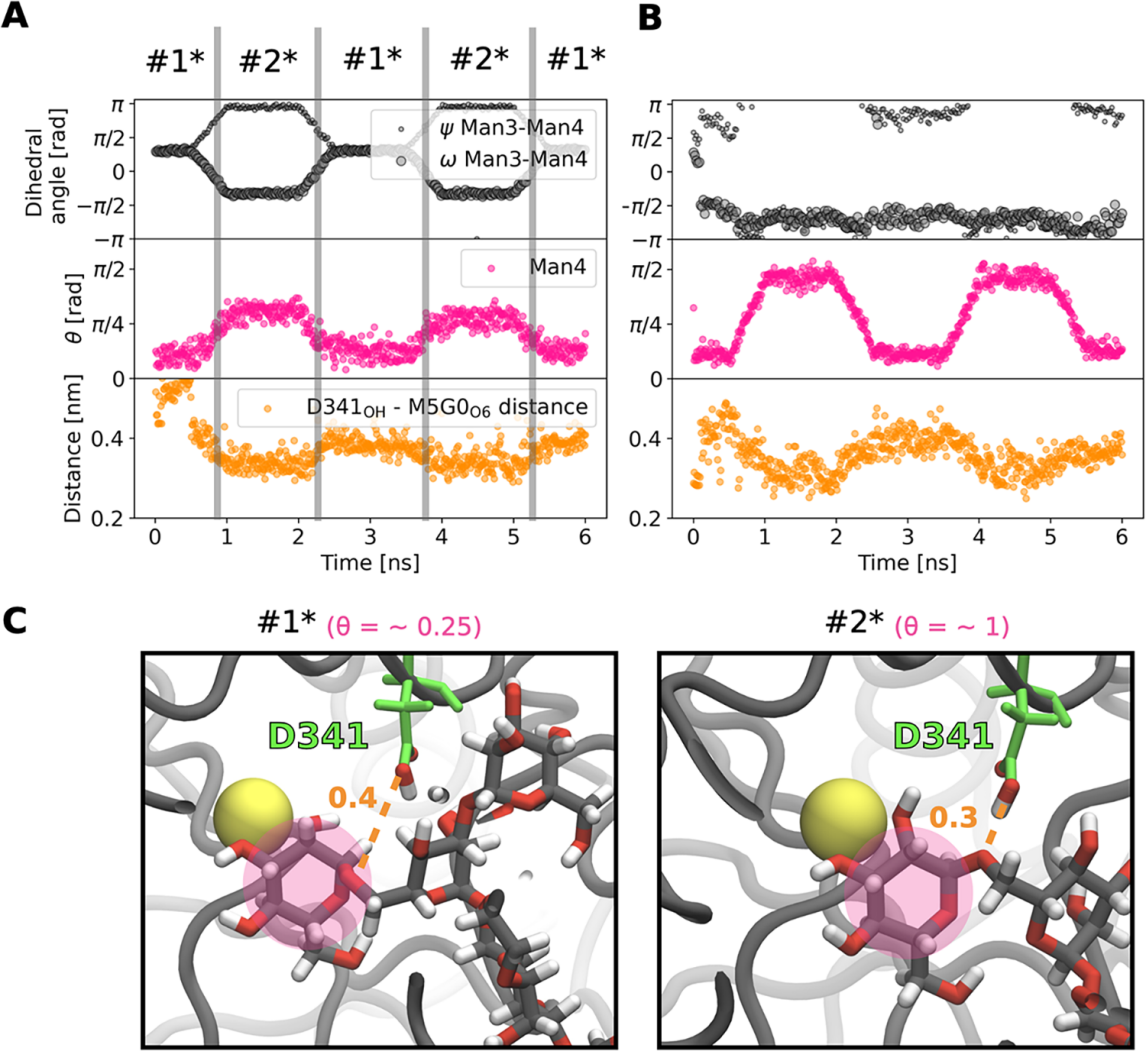
Local conformer #2* induces ring distortion and shortens D341_OH_–M5G0_O6_ bond. Monitoring of selected dihedral angles, ring distortion of Man4 (θ), and D341_OH_–M5G0_O6_ distance over steered MD simulations of native MII where (A) dihedrals of M5G0 are altered to switch between conformers #1*/#2* and (B) θ of Man4 is altered between chair ^4^ *C*_1_ and boat/skew-boat structures. (C) Atomistic snapshots of (A) in the #1* and #2* conformers, respectively, highlighting the difference in D341_OH_–M5G0_O6_ distance associated with reorientation of dihedral angles and therefore conformers. M5G0 is shown in licorice with gray carbon atoms, D341 in green, the Zn^2+^ ion as a yellow sphere, and the protein in cartoon style in dark gray. The subsite −1 is highlighted by a pink circle.

We can thus conclude that the activation of Man4 and formation of the reactant state are the consequence of the following events. First, binding into the catalytic site of MII imposes a constraint on the dihedral-angle conformational phase space of M5G0. Second, the dihedral shift initiates the ring distortion at subsite *–*1, which leads to a reduced D341_OH_–M5G0_O6_ distance, as required by the chemical reaction initiated by a proton transfer from D341 to O6 of M5G0. The simultaneous charge redistribution on the ring further allows for full distortion of Man4 to the ^*O*^ *S*_2_/*B*_2,5_ reactant state, ensuring precise positioning of the scissile glycosidic linkage to be cleaved within the catalytic site.

### The Reactant State: The Factors Governing Enzyme Specificity

The knowledge we have gained about the factors governing the formation of the reactant state can help us shed light on other aspects of MII catalysis. In particular, a comprehensive understanding of the substrate binding is needed to design selective inhibitors for MII, which do not bind to a similar lysosomal α-mannosidase (L-MII).^10^ Previous studies have highlighted that a specific MII region, characterized by amino acids Q64, Y267, H273, P298, W299, and R410 (Figure 7A), plays a crucial role in the binding selectivity of MII toward M5G0. Specifically, the terminal GlcNAc7 residue, which differentiates M5G0 from other high-mannose-type *N*-glycans, binds to this region, which was referred to as the anchor site.^45^ Experiments have shown that the smaller M5 substrate (Figure 7 B), lacking the terminal GlcNAc7 residue, can bind to MII. However, the glycolytic activity of MII toward M5 is reduced by a factor of 80 compared to M5G0, probably due to increased ligand flexibility.^17,45^

**Figure 7:**
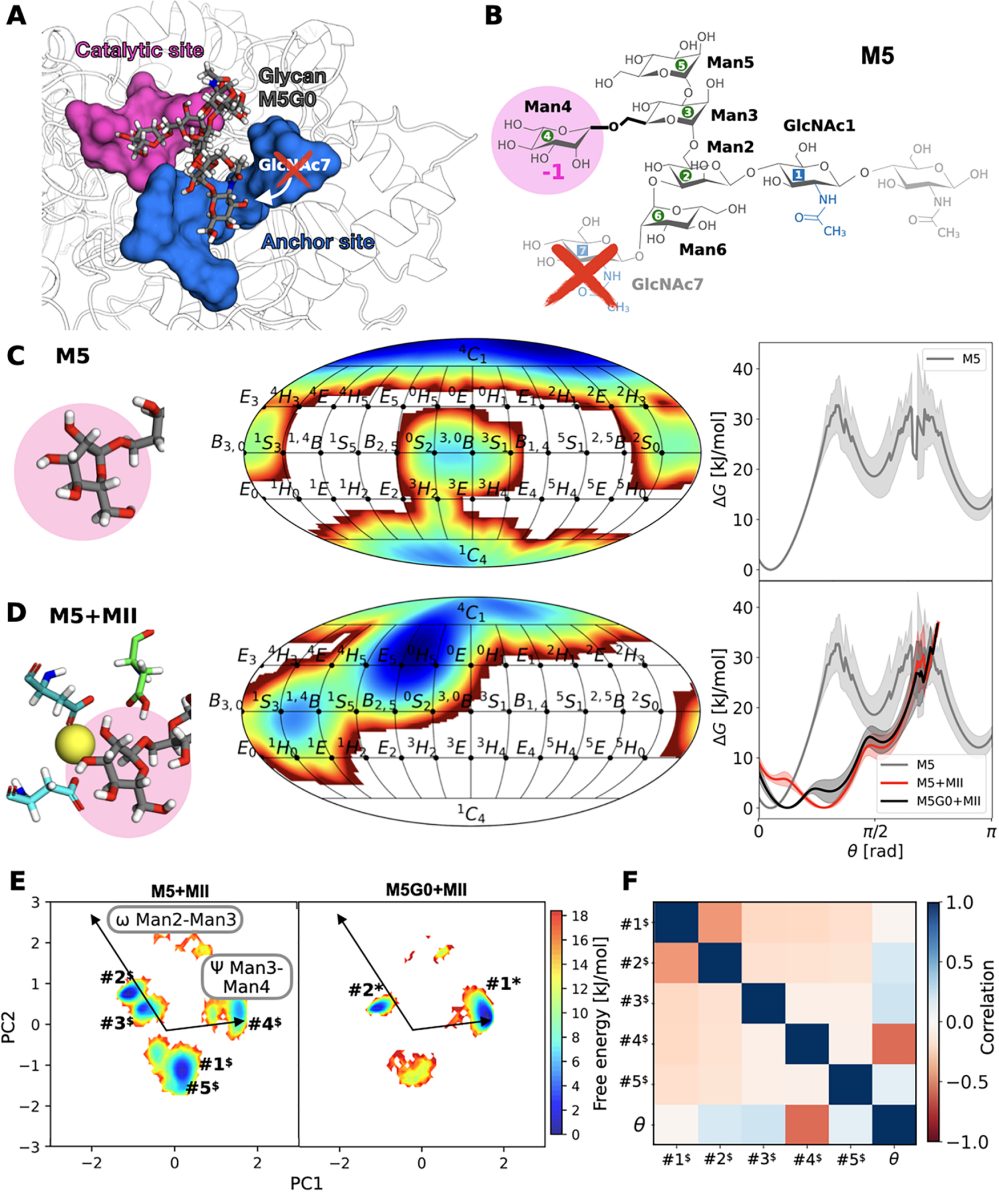
Unoccupied anchor site results in a larger conformational phase space for M5 bound to MII. (A) Atomistic snapshot of catalytic (pink) and anchor site (blue) in MII with bound M5G0. Removal of GlcNAc7 results in M5 and an unoccupied anchor site. (B) Atomistic structure and nomenclature of M5 with the lacking GlcNAc7 shown in transparent and marked by a red cross. (C) Ring distortion of the terminal Man4 in glycan M5 monitored in a 2D representation along ϕ and θ as well as a 1D representation along θ in aqueous solution (gray). (D) Same, for M5 bound to MII (red) compared to the M5G0 + MII system (black). (E) Comparative free energy surfaces of the conformational phase space for M5 + MII and M5G0 + MII projected along PC1 and PC2. (F) Pearson correlation matrix for M5 conformers and θ of Man4 bound to MII.

Quantification of glycan conformers, comparing the conformational phase space of M5 before and after binding to MII, can explain the relationship between anchor site binding and catalytic efficiency (Figure 7).^45^ REST-RECT simulations of M5 + MII revealed a similar ring distortion away from ^4^ *C*_1_ toward ^O^ *H*_5_ as it was found for M5G0 + MII (Figure 7 C,D), indicating that GlcNAc7 in the anchor site is not essential for inducing distortion at subsite −1 (Figures S18 and S19). Yet, the substrate in M5 + MII samples a broader conformational phase space than in M5G0 + MII, with five conformers exceeding a 5% probability (#1^$^– #5^$^ in Figure 7 E). Notably, #4^$^ in M5 + MII corresponds to #1* in M5G0 + MII and shows a negative correlation to the θ puckering of Man4 (Figure 7 F), whereas other conformers show no such correlation. Therefore, increased flexibility of M5 in the binding site of MII seems to prevent optimal Man3-Man4 glycosidic linkage rearrangement for cleavage as the main M5 + MII conformers vary, especially in this linkage. Interestingly, despite differing in only one monosaccharide, the conformational phase spaces of M5 and M5G0 in solution are also significantly different (Figure S20).

As L-MII lacks an anchor site, MII-selective inhibitors could be designed to strongly bind to the anchor site while simultaneously mimicking the #2* conformation of M5G0 in the catalytic site. This dual functionality would ensure tight binding and selectivity toward MII, thus reducing the probability of binding to L-MII.^45^

### Extension to Other CAZymes

The methodology and workflow that we have developed can be seamlessly applied to CAZymes other than MII, enabling a deeper understanding of their own specificity and catalytic mechanisms. As an example, we investigated the related MI enzyme, a GH47 α-mannosidase that cleaves the high-mannose type N-glycan M9 (Figure 8 B). MI has an activate site Ca^2+^ ion, with E599 and E330 as the catalytic base and catalytic acid, respectively (Figure 8 A).^46^ This enzyme is critical for the maturation of *N*-linked oligosaccharides in mammalian cells and participates in the degradation of misfolded glycoproteins.^46–49^ Unlike MII, which targets the flexible α1–6 linkage, MI specifically hydrolyzes the α1–2 glycosidic bond between Man6 and the terminal monosaccharide Man7 at subsite −1 (Figure 8 B).^50^

**Figure 8:**
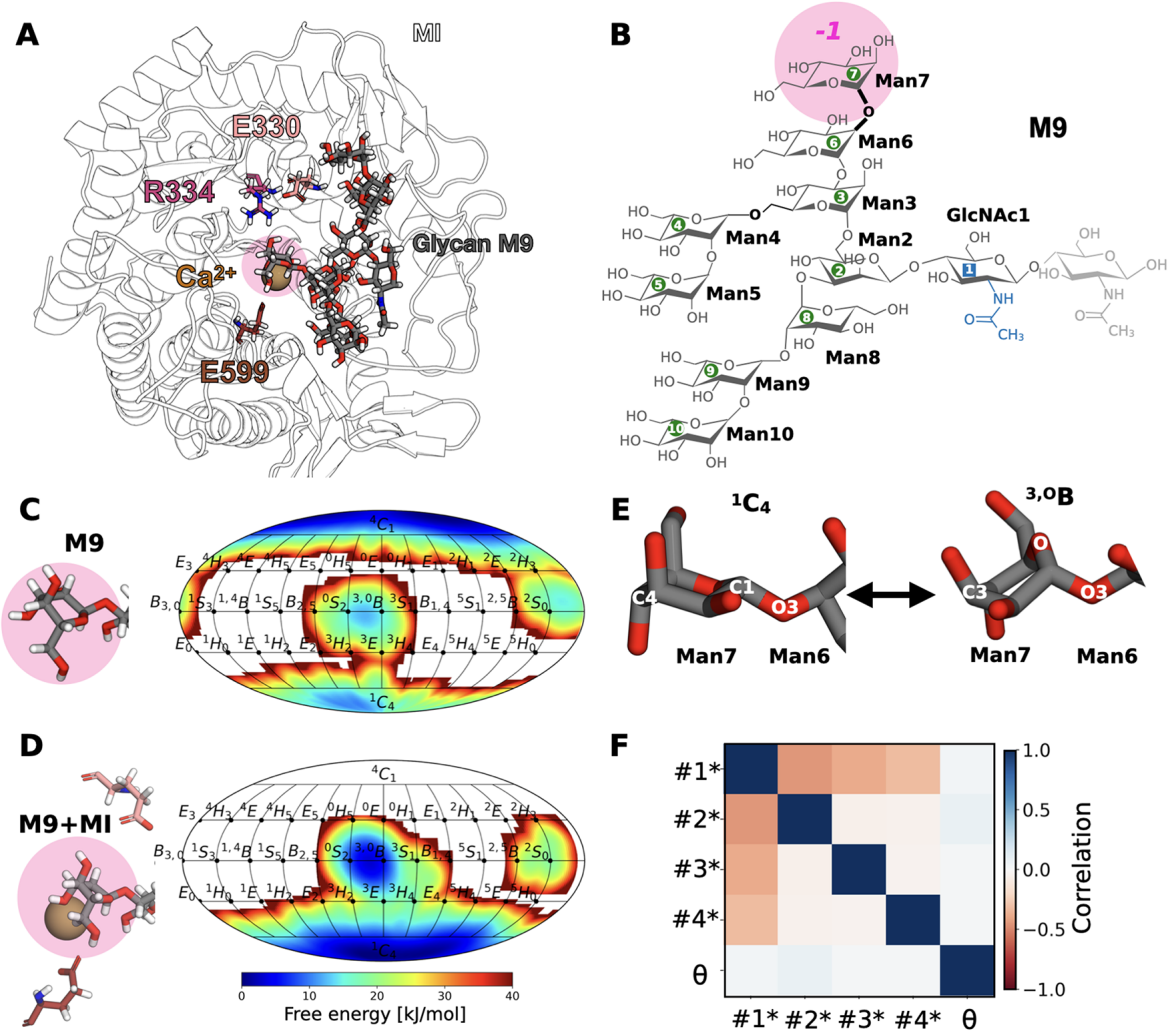
No correlation between ring distortion and glycan conformers is found for M9 in MI. (A) Atomistic structure of MI with bound glycan M9, Ca^2+^ ion, and catalytic residues E330, R334, and E599. (B) Atomistic structure and nomenclature of M9 with labeling of linkages according to the neighboring residue numbers. The Man6-Man7 linkage is highlighted in bold and is to be cleaved by MI. (C) Ring distortion of the terminal Man7 at subsite *–*1 in glycan M9 was monitored in a 2D representation along ϕ and θ in aqueous solution. (D) Same for M9 in the catalytic site of MI. (E) Details of the orientation of the Man6-Man7 glycosidic bond in the two main puckering states. (F) Correlation matrix for M9 conformers and θ of Man7 bound to MI.

Consistently with QM/MM simulations, REST-RECT simulations of M9 before and after binding reveal critical differences in the ring distortion behavior of Man7.^51^ Binding to MI induces a shift from the ^4^ *C*_1_ chair in solution (Figure 8 C) to the ^1^ *C*_4_ chair (Figure 8 D), consistent with experimental observations,^50^ while simultaneously increasing significantly the population of the reactant-state boat ^3,O^ *B*.^51^ Upon binding to MI, the conformational phase space of M9 is notably restricted to one dominant conformer (#1*) with minimal contributions from other conformers (Figures S21–S23). Importantly, the glycosidic bond between Man6 and Man7, formed via the O3 atom, remains in the same conformation regardless of ring distortion and the glycan conformer (Figure 8 E). This is further supported by the absence of correlation between ring distortion in Man7 and the glycosidic bond orientation for all conformers (Figure 8F). Therefore, our finding shows that correlations between dihedral angles and puckering degrees of freedom are CAZyme-specific and not required where structural rearrangements of the bond to be cleaved are unnecessary.

## Conclusions

The formation of the reactant state in the catalytic itinerary of glycosidases is the first step toward glycosidic bond hydrolysis and often includes ring distortion of the monosaccharide at subsite −1. The origin of this distortion was investigated by enhanced-sampling molecular dynamics simulations with the REST-RECT methodology, providing quantitative structural insights into the formation of the reactant state in mannosidases. Comparing three different substrates and two different enzymes, we demonstrated that the essential chair-to-boat ring distortion at subsite −1 is not achieved by a universal mechanism and is strongly influenced by the dynamics of the glycan substrate.

We were able to effectively sample and quantify the restricted conformer distribution of bound glycan substrates, which, although very enzyme-dependent, is altered by small modifications of the substrate, such as the absence of one terminal saccharide in M5 with respect to M5G0, or of the enzyme, such as the mutation of single amino acids in the catalytic site. Specifically, MI locks its substrate M9 in only one global conformer with puckering alternating between ^1^ *C*_4_ and ^3,O^ *B*, whereas the substrate of MII, M5G0, can adopt two distinct conformers with distinct pucker states ^4^ *C*_1_ and ^O^ *H*_5_. In both cases, the puckers differ substantially from those adopted in solution. Upon binding, the terminal monosaccharide at subsite −1 increases its propensity to distorted ring puckering in all of the studied substrate/enzyme systems. In M5G0 bound to MII, the ring distortion is strictly correlated with the glycan adopting a certain global conformation, determined by the dihedral angles of the glycosidic bonds. This uncovers a previously unrecognized relationship between dihedral angles and puckering degrees of freedom, which in turn promotes an orientation of the glycosidic bond favorable for hydrolytic cleavage. This correlation is not observed for M9 bound to MI, as only one conformer is adopted in the binding pocket with an already favorable orientation of the to-be-cleaved glycosidic bond.

Our findings emphasize the need for accessing the full conformational space, rather than determining only static snapshots, when attempting to fully understand the specificity and catalytic efficiency of CAZyme/substrate complexes. Although the employed REST-RECT sampling approach has shown reproducible conformer population distributions for glycans in solution in earlier studies, one must note the greatly increased difficulty of conformer sampling for glycan-enzyme complexes. Full convergence could be hindered by increased barrier heights for the rotation around glycosidic bonds due to strong molecular interactions between amino acid and glycan residues, artificially stabilizing or destabilizing certain conformations that lead to incorrect population distributions. In addition, it is important to note the limited validation of the force field parameters for the correct equilibrium distribution of the glycan conformations. There exist only a few experimental values, e.g., NMR J-coupling constants, for comparison of dihedral angle behavior and ring-conformational equilibria.^3,32^ Despite the adoption of ring distorted shapes in the GLYCAM06j force field, a lack of polarization prevents the accurate reproduction of ring distortions comparable to QM/MM results and requires the testing of more advanced force field types in the future.^16^

The methodology presented in this study provides a computational framework for the rational advancement of drug development strategies aimed at modulating CAZyme activities. CAZyme inhibitors are currently designed by aiming for precise saccharide conformations that resemble certain states in the catalytic itinerary of substrates, particularly in cancer treatment and other glycosylation-related diseases. Here we found that, in addition to designing relatively large drug molecules that simultaneously bind both to the anchor site and the catalytic site of MII, the specific #2* conformation could enhance an efficient inhibition of MII while minimizing a concomitant unwanted binding to L-MII.^10^ As different CAZyme families feature different mechanisms, further tailored studies are needed that take into account the unique structural and chemical features of the individual enzymes and substrates.

## Supplementary Material



## Data Availability

The generation of glycan conformer strings and conformer distribution plots was done by GlyCONFORMER, a Python-based package deposited on GitHub under https://github.com/IsabellGrothaus/GlyCONFORMER. PLUMED input files for REST-RECT and steered MD simulations were deposited in the PLUMED-NEST repository under PlumID:25.007. Structure and trajectory files for all simulations can be accessed from 10.5281/zenodo.14858832. For REST-RECT simulations, only the ground replica trajectories are provided.

## Footnotes

1 Carbohydrate-Active Enzymes database accessed ninth December 2024, http://www.cazy.org

## Notes

The SI provides GlyCONFORMER analyses for glycans simulated either in solution or bound to one of the CAZymes, including the glycan conformer distribution, convergence of glycan conformers during REST-RECT simulations and latent space projection via PCA of the conformer distribution. Furthermore, it provides additional plots for important protein-glycan distances and 2D free energy profiles of conformer distributions for several simulation systems. Detailed correlation matrices of dihedral angles and pucker coordinates are provided for M5G0 and M5G0 + MII (PDF)
