# Correction to “Shaping the Glycan Landscape: Hidden Relationships between Linkage and Ring Distortions Induced by Carbohydrate-Active Enzymes”

**DOI:** 10.1021/jacs.6c03061

**Published:** 2026-03-13

**Authors:** Isabell Louise Grothaus, Paul Spellerberg, Carme Rovira, Lucio Colombi Ciacchi

There are errors in the graphical representation of several mannose units in a number of figures. Specifically, some carbohydrate structures were inadvertently drawn as the l-enantiomer instead of the naturally occurring d-mannose, and in one case an anomeric bond was not depicted with the correct orientation. In Figure 1A, both mannose units were the l-enantiomer mirror image of the natural D-mannose. In Figures 2C and 7B, Man3, Man4 and Man5 were all l-enantiomers. Man4 was depicted in an equatorial bond, but required an axial bond for the alpha anomer. In Figure 8B, Man3, Man4, Man5, Man6, Man9 and Man10 were all depicted in the wrong enantiomer. These errors are limited exclusively to the drawings of the structures, and no simulations and corresponding results and conclusions are affected by the following corrected figures. We would like to thank Dr. Sebastien Vidal for spotting these errors.

**Figure 1:**
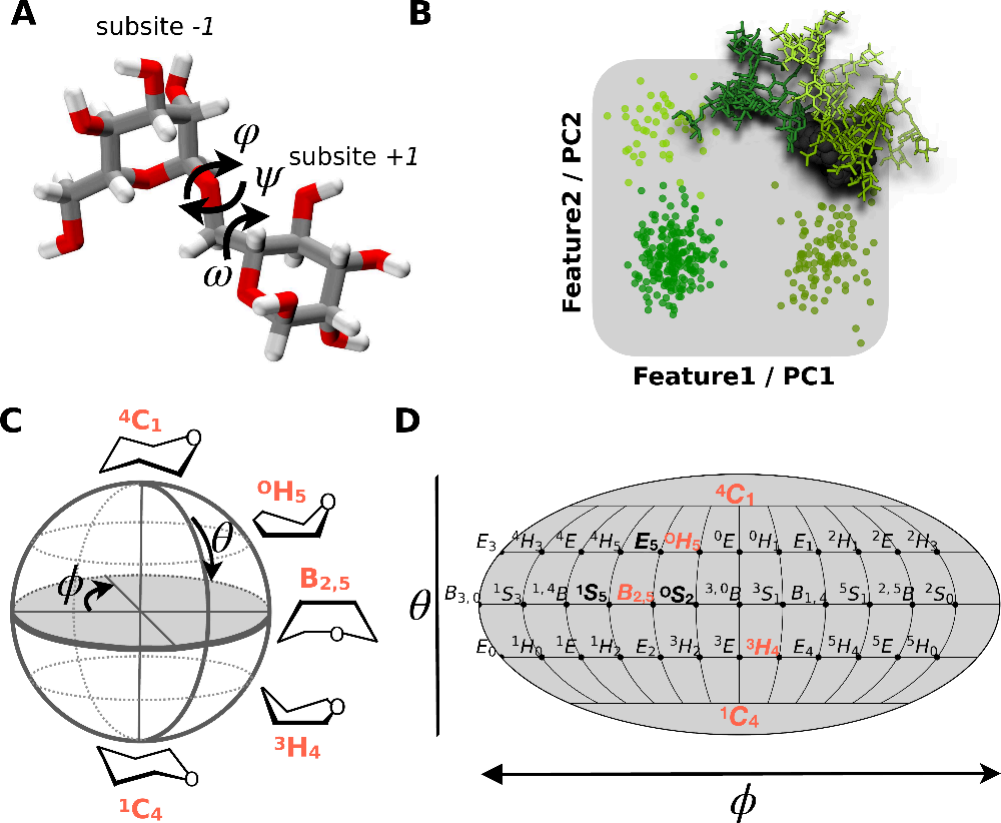
Representation of the glycan’s conformational phase space. (A) Glycans are flexible molecules, adopting various distinct conformers that are characterized by the dihedral angles φ, ψ and ω along the glycosidic bonds connecting the individual monosaccharides e.g. mannoses located at subsite *-1* and *+1* in a CAZyme binding site. The atomistic structure is represented in licorice style with oxygen atoms in red, hydrogens in white and carbons in gray. (B) The conformational phase space can be best represented in low dimensions employing features as axis that can be identified by e.g. principle component analysis. Points in this latent space represent individual structures that are colored by their respective conformer. Atomistic structures colored in green represent three different conformers of the glycan M5G0. (C) An additional degree of freedom is the flexibility of the pyranose ring, whose distortion is quantified by the Cremer-Pople pucker coordinates θ and ϕ and (D) plotted using a Mollweide projection, a pseudocylindrical map with equal-area, meaning that areas, densities and, thus, free energy values are preserved. Labels represent individual ring shapes.

**Figure 2:**
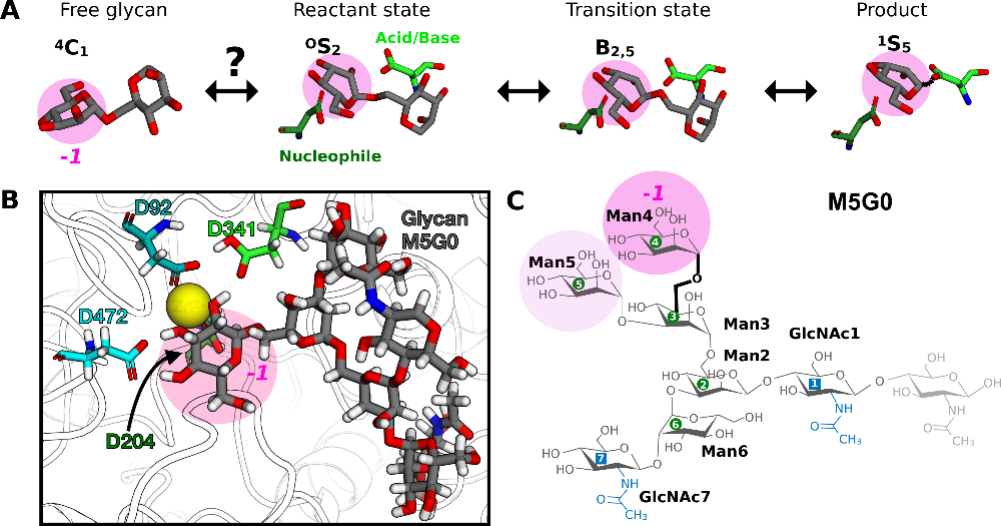
Catalytic details of Golgi α-mannosidase II. (A) The catalytic itinerary of the glycosylation step, depicting the transition from the solution equilibrium ^4^ *C*_1_ chair to the product in the ^1^ *S*_5_ skew-boat. The monosaccharide at subsite *-1* is the residue to be cleaved off. (B) The binding configuration of glycan M5G0 within the catalytic site, highlighting the Zn^2+^ ion and key amino acid residues (D92, D204, D341, and D472). Atoms are displayed in a licorice style: the glycan has gray carbon atoms, while amino acid carbons are color-coded by residue type. Oxygen atoms are red, nitrogen atoms are blue, and hydrogen atoms are white. (C) Atomistic structure and nomenclature of M5G0 with labeling of linkages according to neighboring residue numbers. The Man3-Man4 linkage is highlighted in bold and to be cleaved by MII. In all panels, the to-be-cleaved Man4, positioned at the catalytic subsite *-1*, is emphasized with a transparent pink circle.

**Figure 7:**
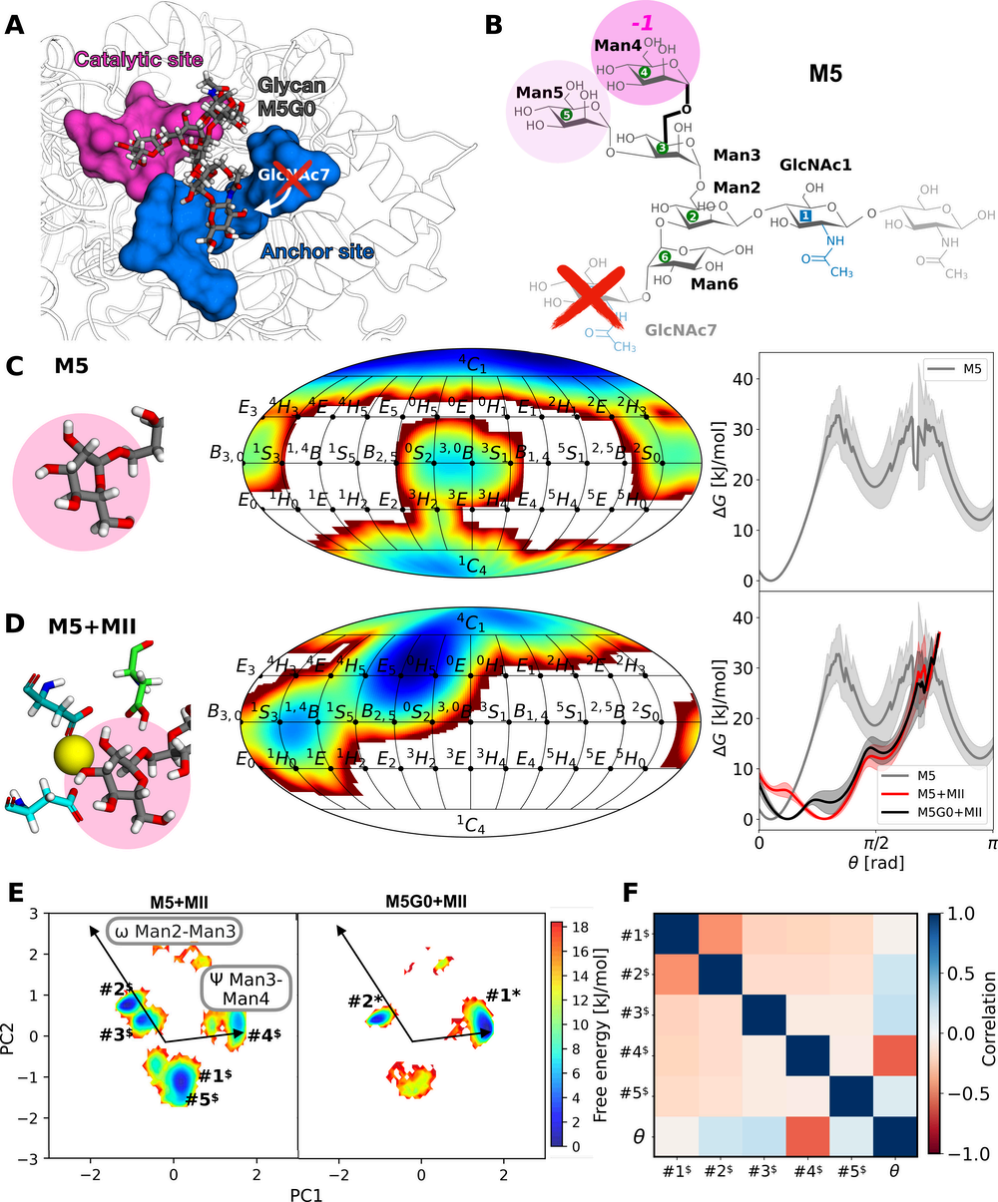
Unoccupied anchor site results in larger conformational phase space for M5 bound to MII. (A) Atomistic snapshot of catalytic (pink) and anchor site (blue) in MII with bound M5G0. Removal of GlcNAc7 results in M5 and an unoccupied anchor site. (B) Atomistic structure and nomenclature of M5 with the lacking GlcNAc7 shown in transparent and marked by a red cross. (C) Ring distortion of the terminal Man4 in glycan M5 monitored in a 2D representation along ϕ and θ as well as 1D along θ in aqueous solution (gray). (D) Same, for M5 bound to MII (red), compared to the M5G0+MII system (black). (E) Comparative free energy surfaces of the conformational phase space for M5+MII and M5G0+MII projected along PC1 and PC2. (F) Pearson correlation matrix for M5 conformers and θ of Man4 bound to MII.

**Figure 8:**
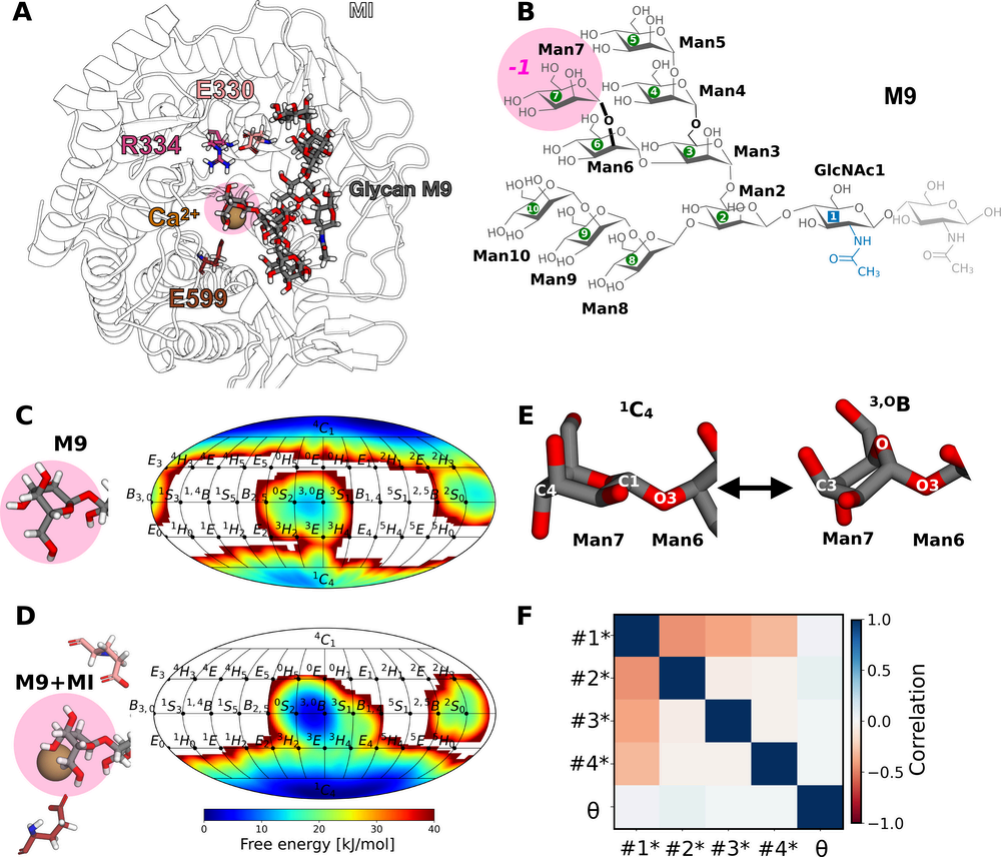
No correlation between ring distortion and glycan conformers is found for M9 in MI. (A) Atomistic structure of MI with bound glycan M9, Ca^2+^ ion and catalytic residues E330, R334 and E599. (B) Atomistic structure and nomenclature of M9 with labeling of linkages according to neighboring residue numbers. The Man6-Man7 linkage is highlighted in bold and is to be cleaved by MI. (C) Ring distortion of the terminal Man7 at subsite *-1* in glycan M9 monitored in a 2D representation along ϕ and θ in aqueous solution. (D) Same, for M9 in the catalytic site of MI. (E) Details of the orientation of the Man6-Man7 glycosidic bond in the two main puckering states. (F) Correlation matrix for M9 conformers and θ of Man7 bound to MI.

