# Supplementary material for: Shaping the Glycan Landscape: Hidden Relationships between Linkage and Ring Distortions Induced by Carbohydrate-Active Enzymes
Source: J Am Chem Soc. 2025 Oct 9;147(42):38376–89. doi: 10.1021/jacs.5c11504 (PMC12550843; doi:10.1021/jacs.5c11504)
Supplement: Supplementary file 1 [file ja5c11504_si_001.pdf]

Supporting Information: Shaping the Glycan Landscape: Hidden relationships between linkage and ring distortions induced by carbohydrate-active enzymes

Isabell Louise Grothaus,[*,†,‡,¶] Paul Spellerberg,[§] Carme Rovira,[‖] and Lucio Colombi Ciacchi[†]

†*Hybrid Materials Interfaces Group, Bremen Center for Computational Materials Science and MAPEX Center for Materials and Processes, University of Bremen, 28359 Bremen, Germany*

‡*Malopolska Centre of Biotechnology, Jagiellonian University, 31-007 Krakow, Poland*

¶*Department of Theoretical Biophysics, Max Planck Institute for Biophysics, 60438 Frankfurt, Germany*

§*Faculty for Biology and Chemistry, University of Bremen, 28359 Bremen, Germany*

‖*Departament de Química Inorgànica i Orgànica & IQTCUB, Universitat de Barcelona, Barcelona 08028, Spain; Institució Catalana de Recerca i Estudis Avançats (ICREA), Barcelona 08020, Spain*

E-mail: grothaus@uni-bremen.de

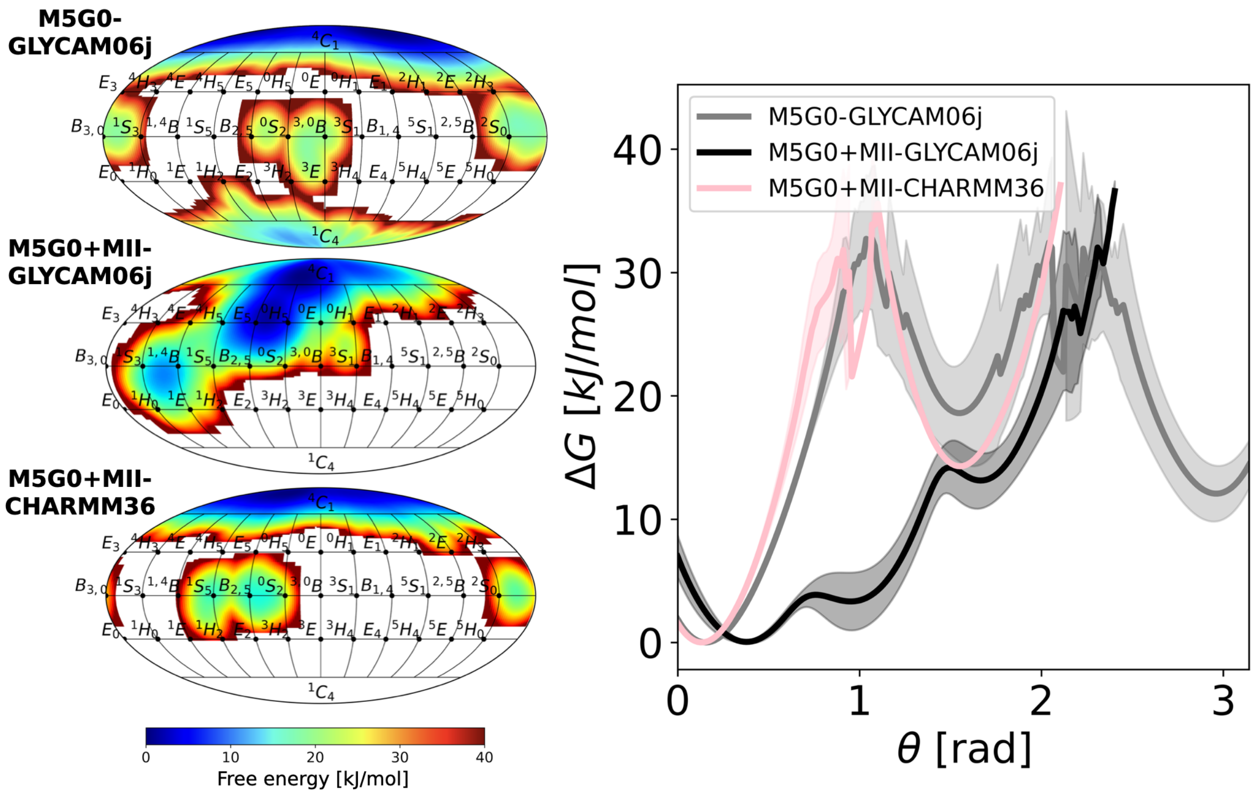

Figure S1: Ring distortion of the terminal Man4 of M5G0 monitored by the 2D Cremer-Pople representation along ϕ and θ as well as 1D along θ, simulated in solution or bound to MII, using different force fields.

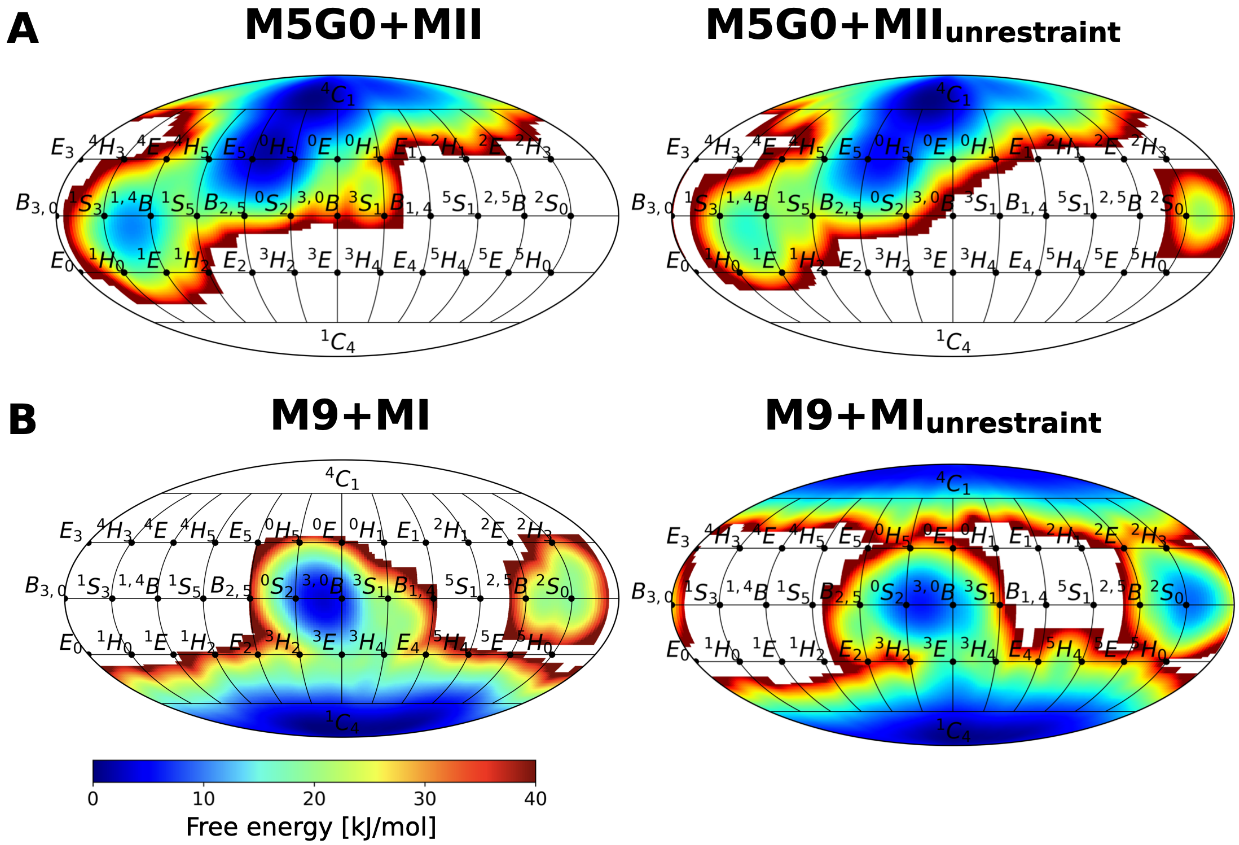

Figure S2: Comparison of ring distortion for **A** M5G0+MII and **B** M9+MI with and without restraints of the substrates to the binding sites. The population of the $^4C_1$ chair conformation in M9+MI$_{\text{unrestraint}}$ comes from snapshots in the trajectory where the glycan unbound and underlines the necessity for fixing the substrates to the catalytic site via distance restraints.

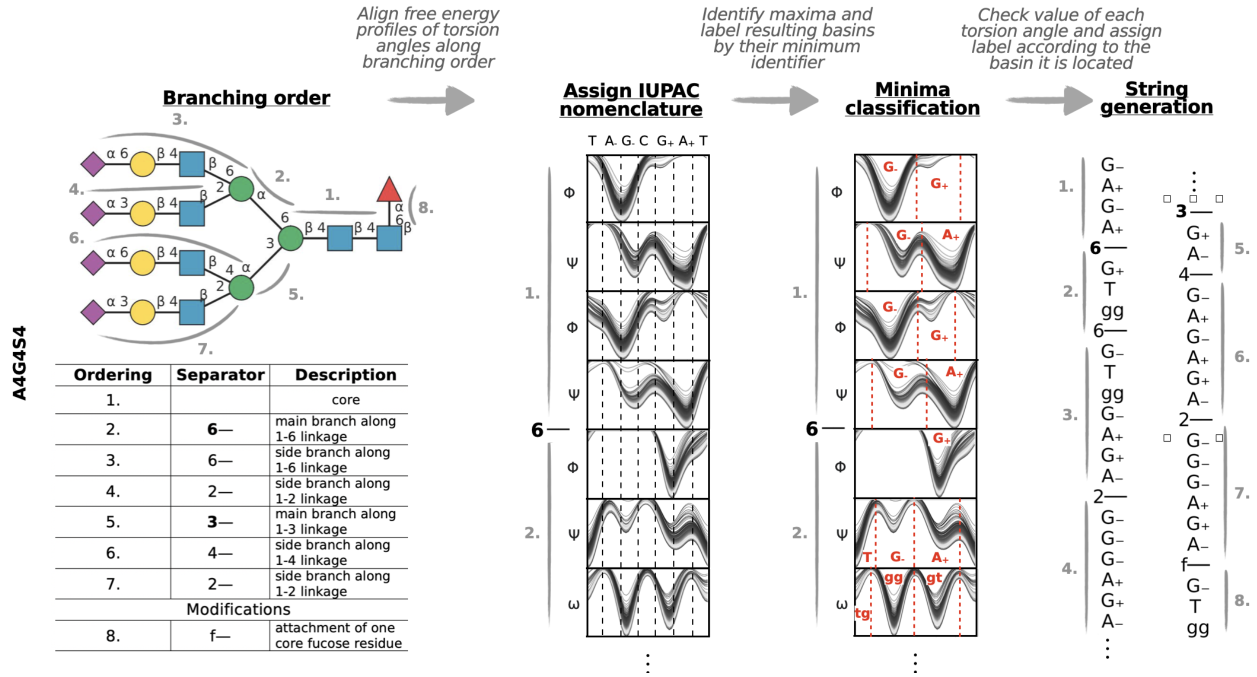

Figure S3: GlyCONFORMER string construction based on linkage dihedral angles for the model glycan A4G4S4.

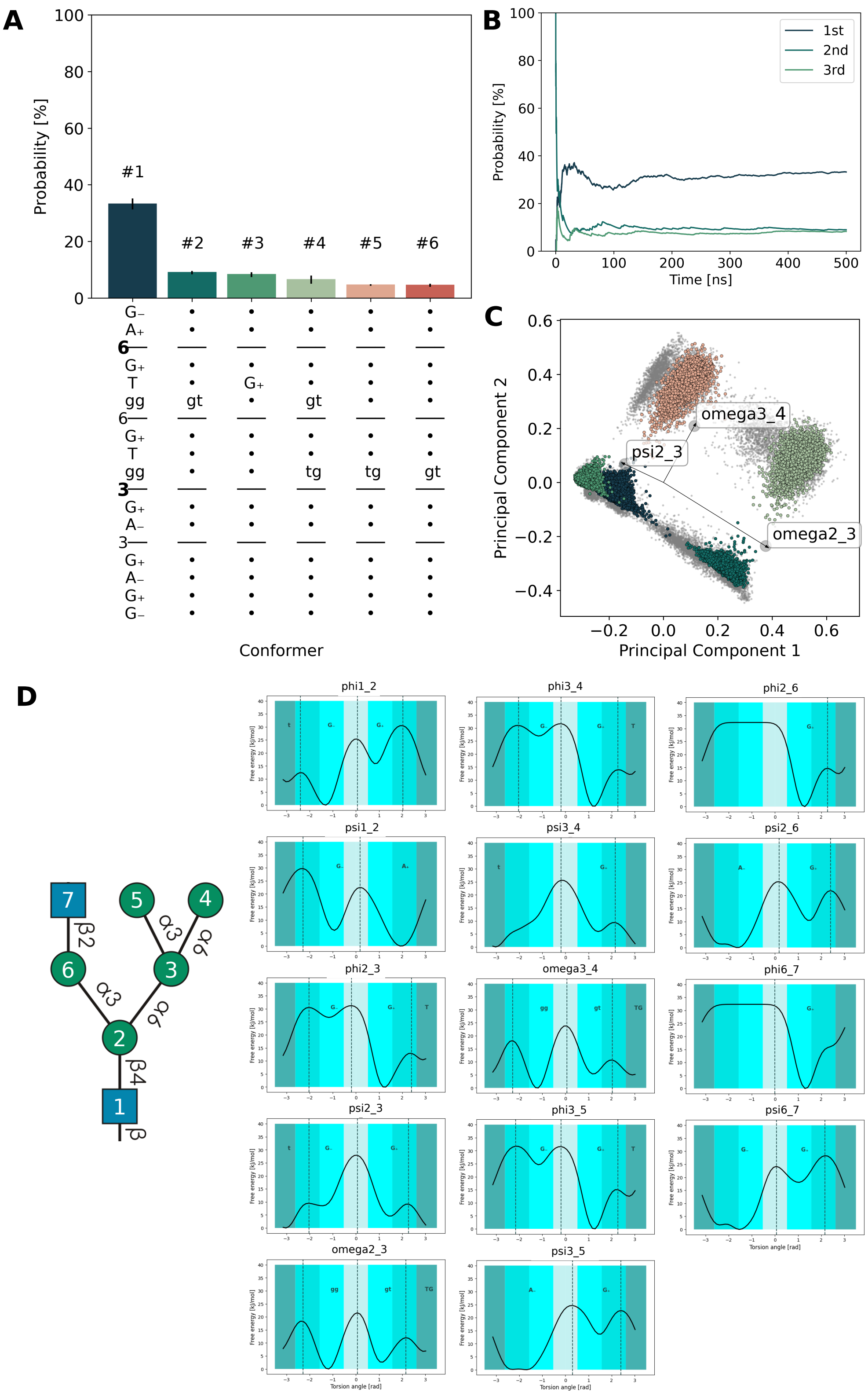

A
B
C
D
Probability [%]
#1
#2
#3
#4
#5
#6
Conformer
1st
2nd
3rd
Time [ns]
Principal Component 1
Principal Component 2
omega3_4
psi2_3
omega2_3
phi1_2
phi3_4
phi2_6
psi1_2
psi3_4
psi2_6
phi2_3
omega3_4
phi6_7
psi2_3
phi3_5
psi6_7
omega2_3
psi3_5
Free energy [kJ/mol]
Torsion angle [rad]

Figure S4: GlyCONFORMER[S1] analysis of M5G0 in solution displaying the conformer distribution as a histogram with each conformer representing one bin **A**, the cumulative average of the three most dominant conformers **B** and the conformational phase space of **A** represented in two dimensions via PCA, using all dihedral angles as input features. Vectors indicate the original feature axes with highest variance, where they point in the direction with highest squared multiple correlation with the principle components. **D** Free energy profiles sampled and reconstructed from REST-RECT simulations for each dihedral angle occurring in the glycan structure and lined up in the order as they occur in the GlyCONFORMER string. Plots are labeled by the type of dihedral angle, where the numbers correspond to the monosaccharide residue numbers from the glycan scheme on the left. Letters label each minima, whose boundaries are defined by surrounding energy barriers that are marked by vertical dashed lines. Shaded regions display the IUPAC defined ranges of torsion angles.

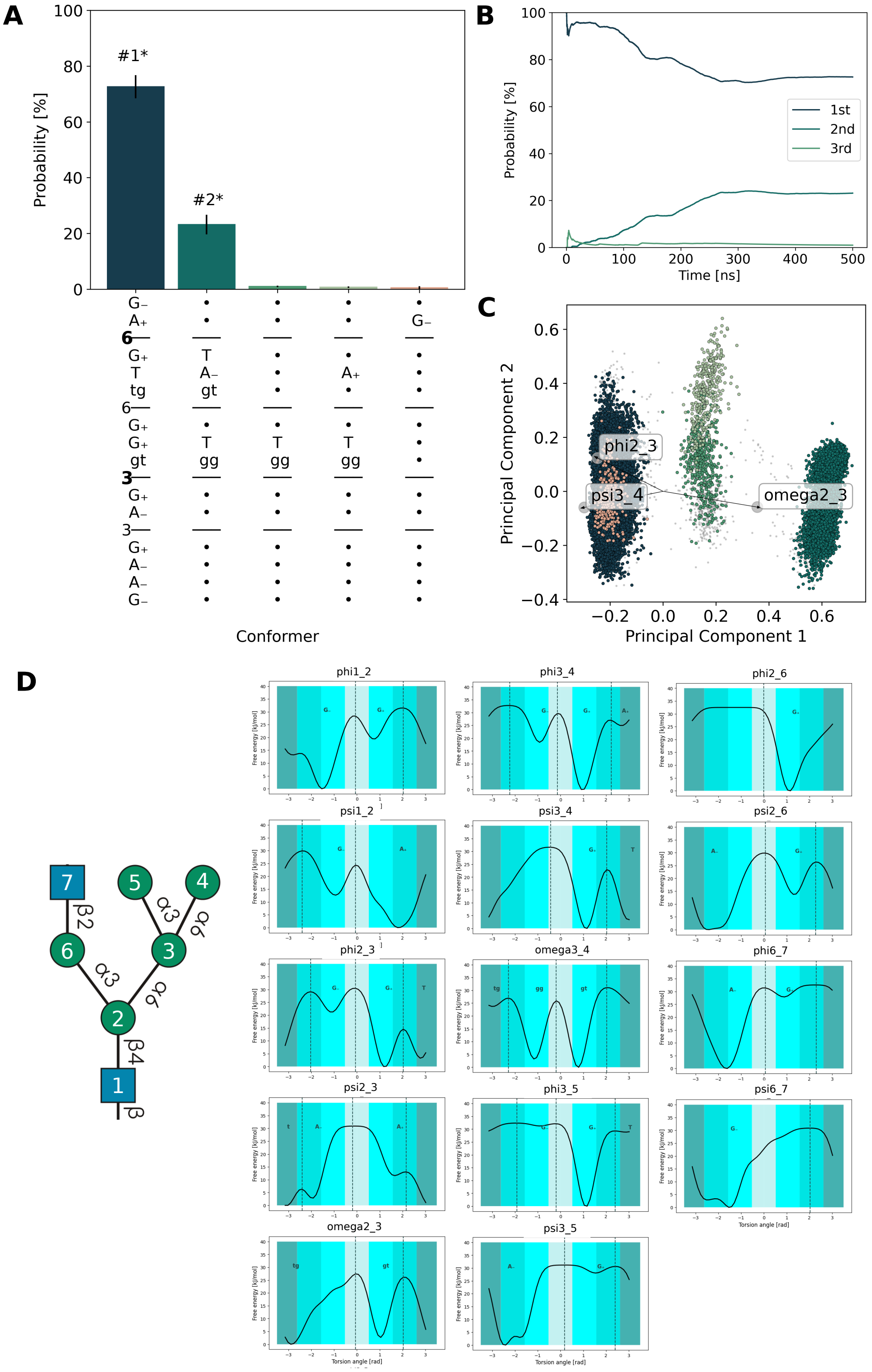

Figure S5: GlyCONFORMER analysis of M5G0 bound to MII with same panels as in Figure S4.

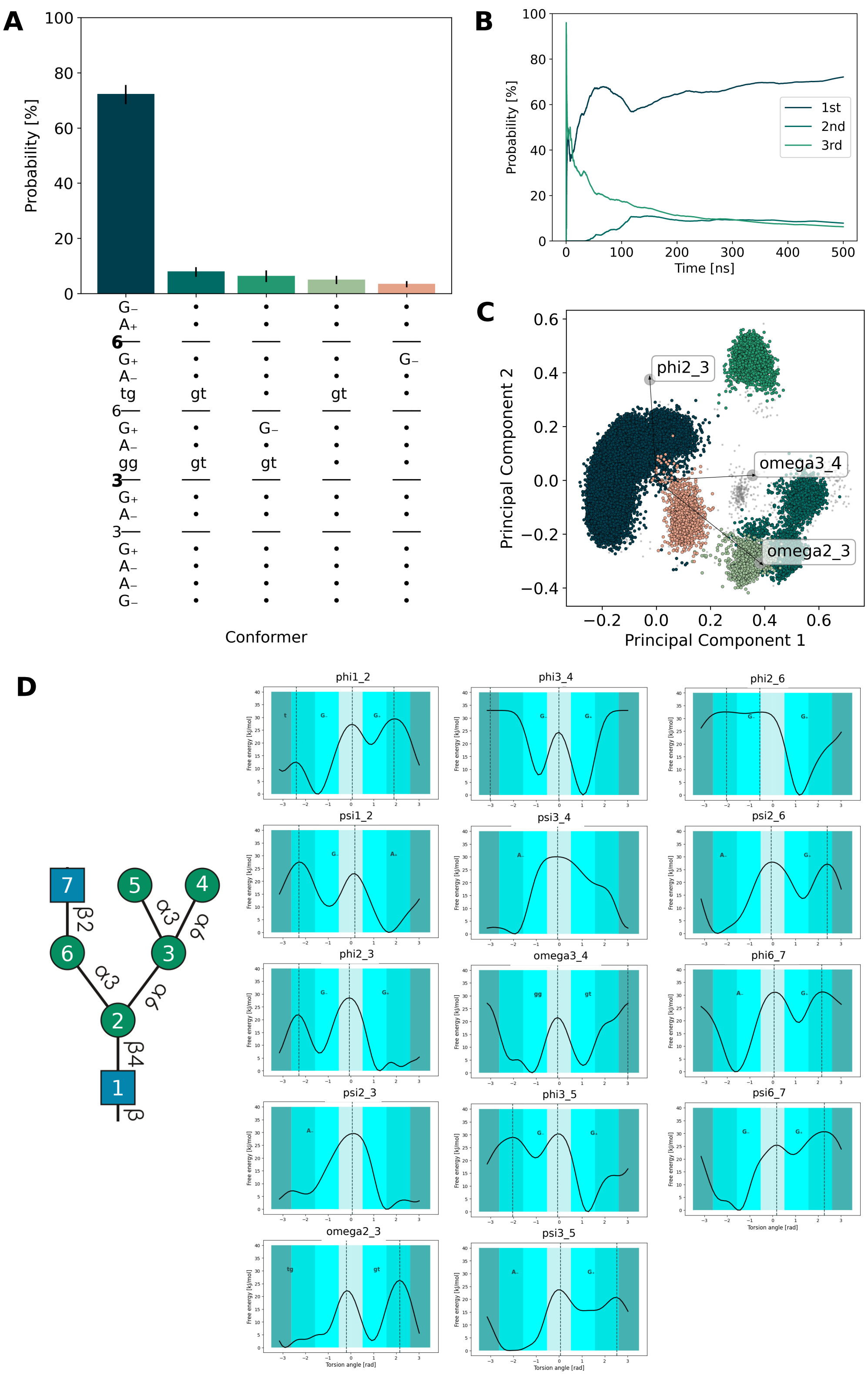

Figure S6: GlyCONFORMER analysis of M5G0 bound to MII with a restrain on the D341$_{\mathrm{H}}$ - M5G0$_{\mathrm{O6}}$ distance between amino acids D341 and the oxygen of the glycosidic linkage to be cleaved. Same panels as in Figure S4.

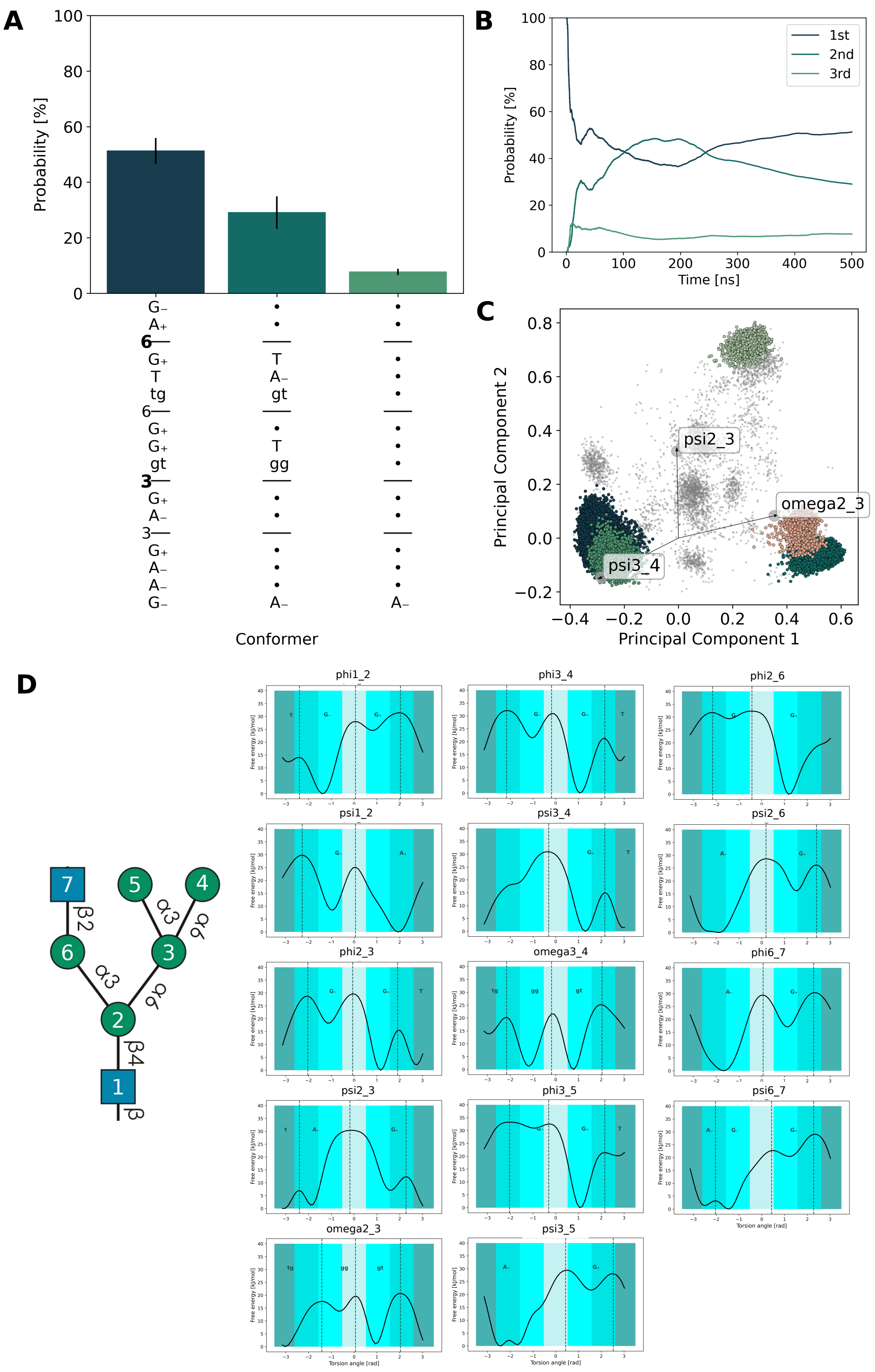

Figure S7: GlyCONFORMER analysis of M5G0 bound to mutant D204A MII with same panels as in Figure S4.

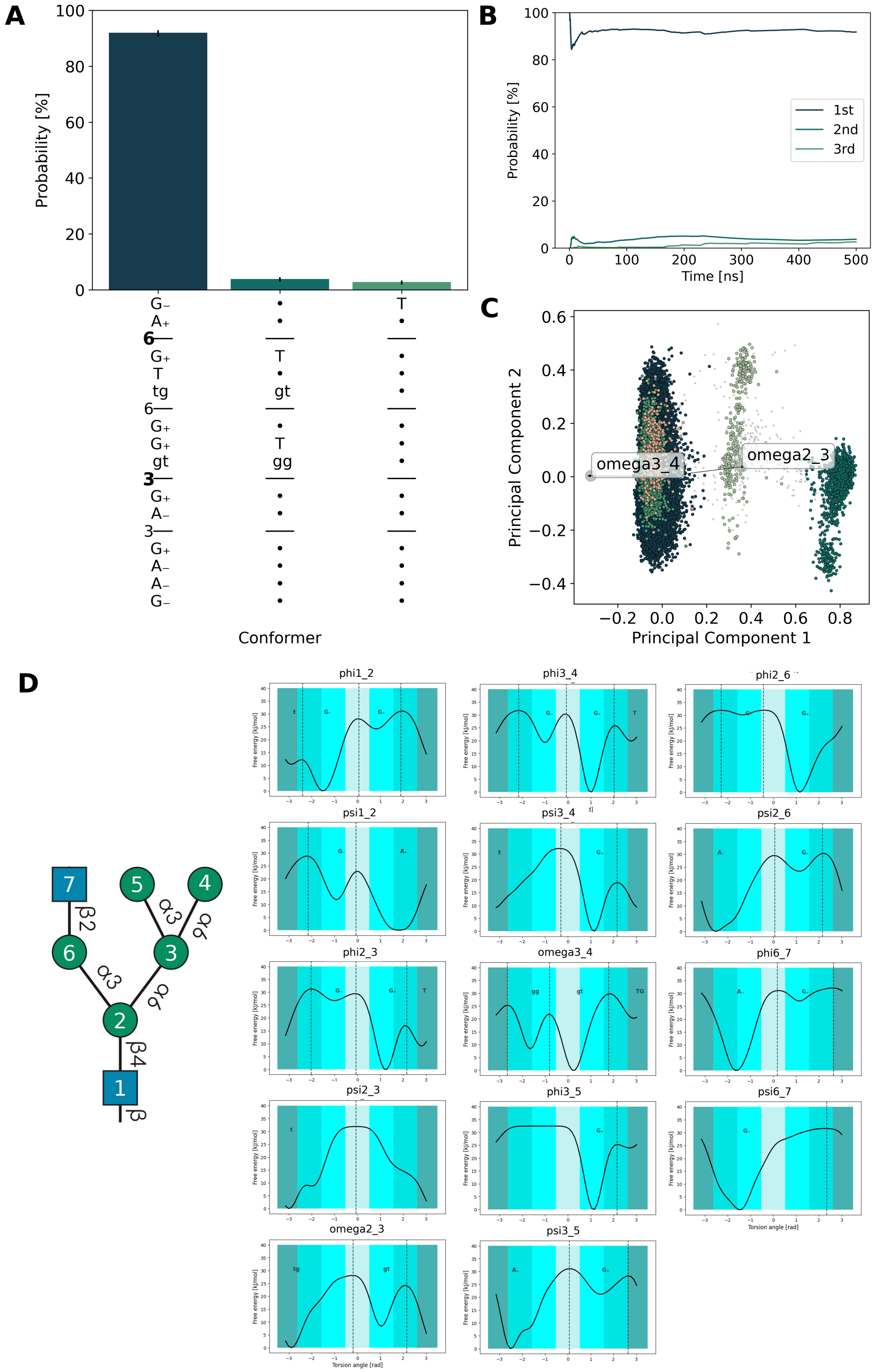

Figure S8: GlyCONFORMER analysis of M5G0 bound to mutant D341A MII with same panels as in Figure S4.

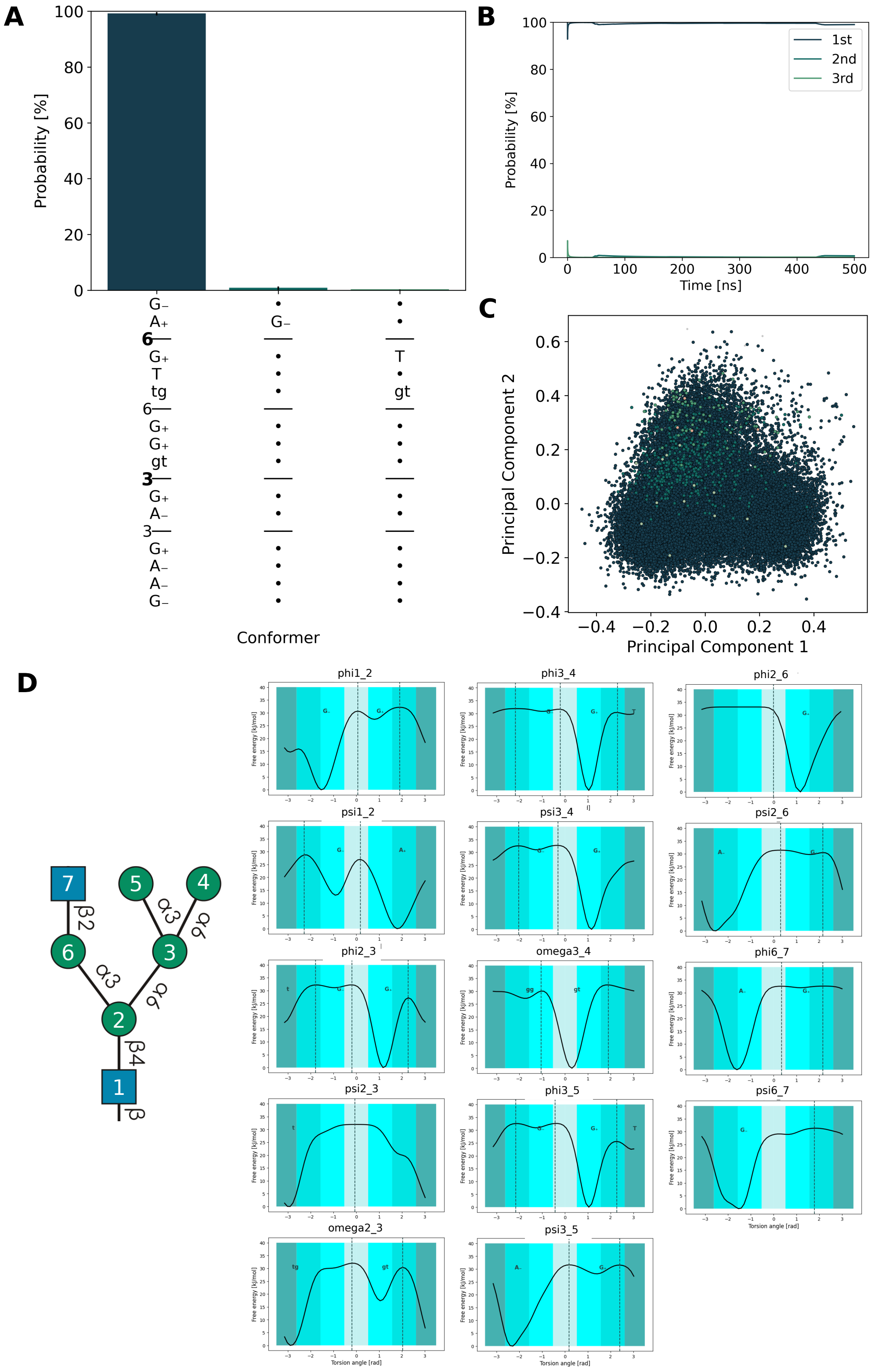

Figure S9: GlyCONFORMER analysis of M5G0 bound to mutant D92A MII with same panels as in Figure S4.

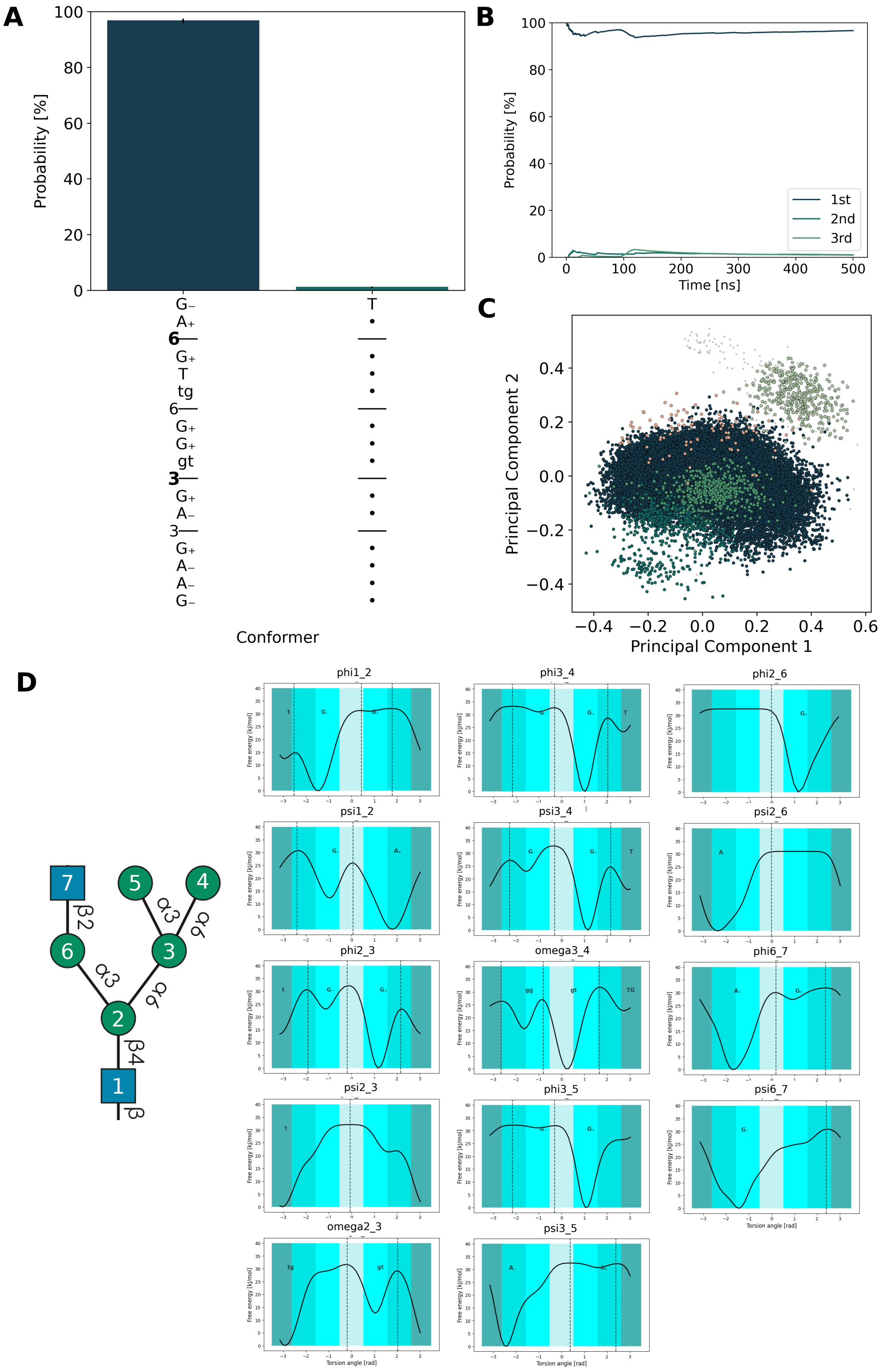

Figure S10: GlyCONFORMER analysis of M5G0 bound to mutant D472A MII with same panels as in Figure S4.

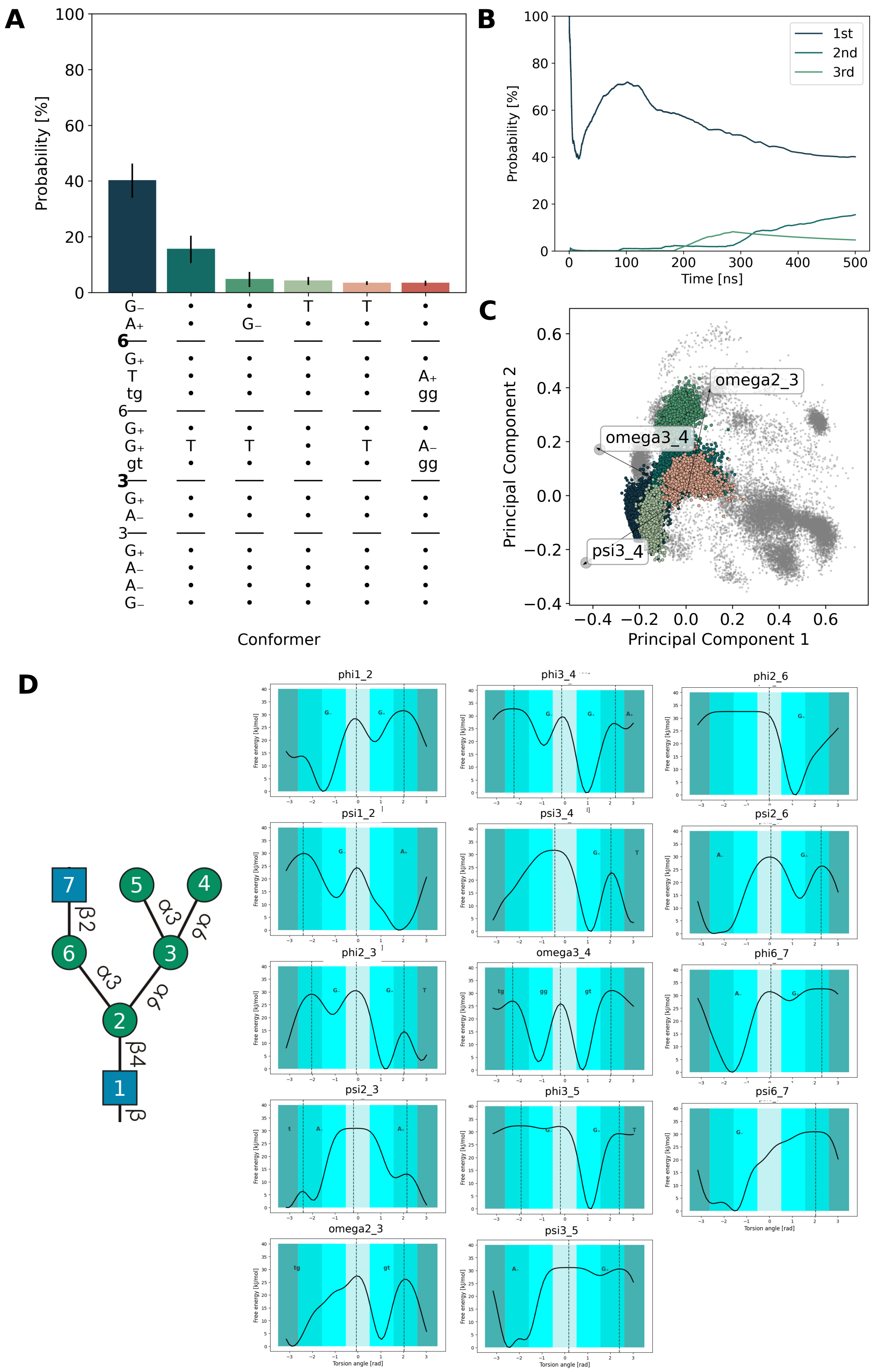

Figure S11: GlyCONFORMER analysis of M5G0 bound to MII lacking the Zn ion with same panels as in Figure S4.

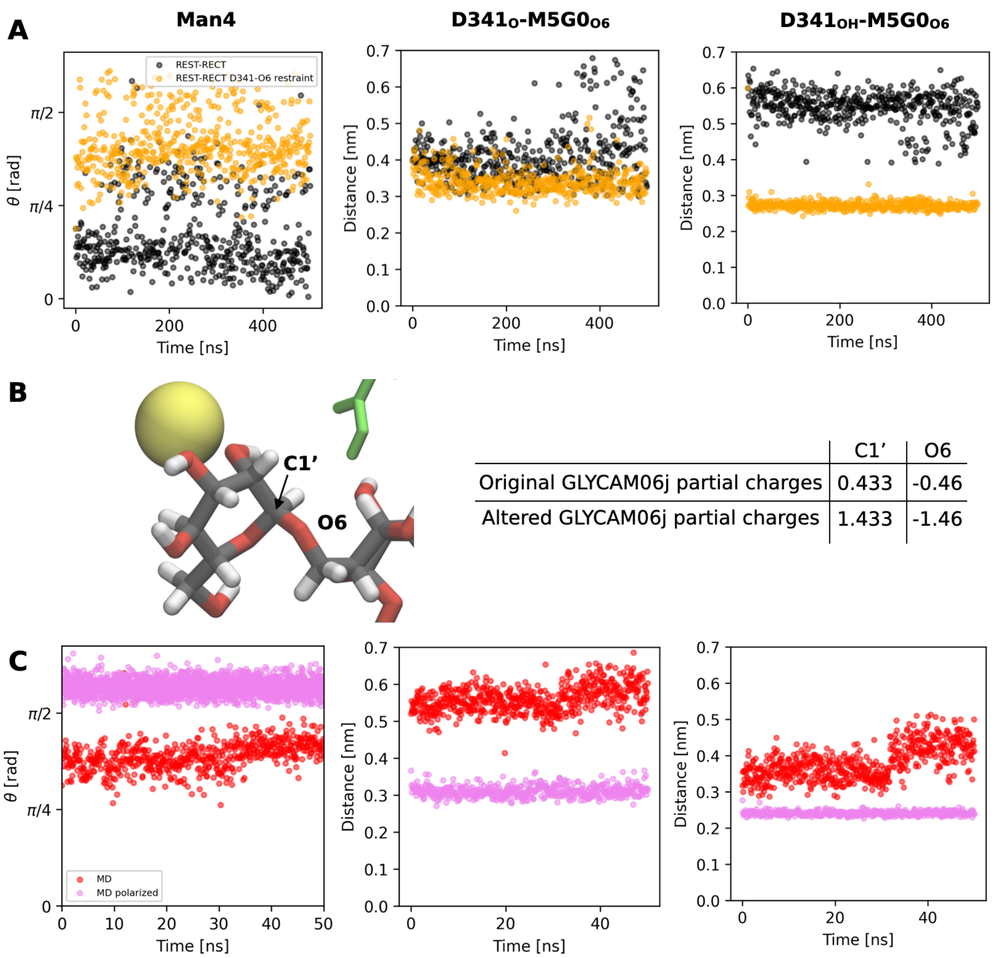

	C1’	O6
Original GLYCAM06j partial charges	0.433	-0.46
Altered GLYCAM06j partial charges	1.433	-1.46

Figure S12: Ring distortion captured along θ and distances D341$_{OH}$ - M5G0$_{O6}$ and D341$_{O}$ - M5G0$_{O6}$ recorded for **A** REST-RECT simulations of M5G0+MII and M5G0+MII-D341$_{H}$ - M5G0$_{O6}$, **C** conventional MD simulations of M5G0+MII and M5G0+MII-polarized with altered partial charges on C1’ and O6 as described in **B**.

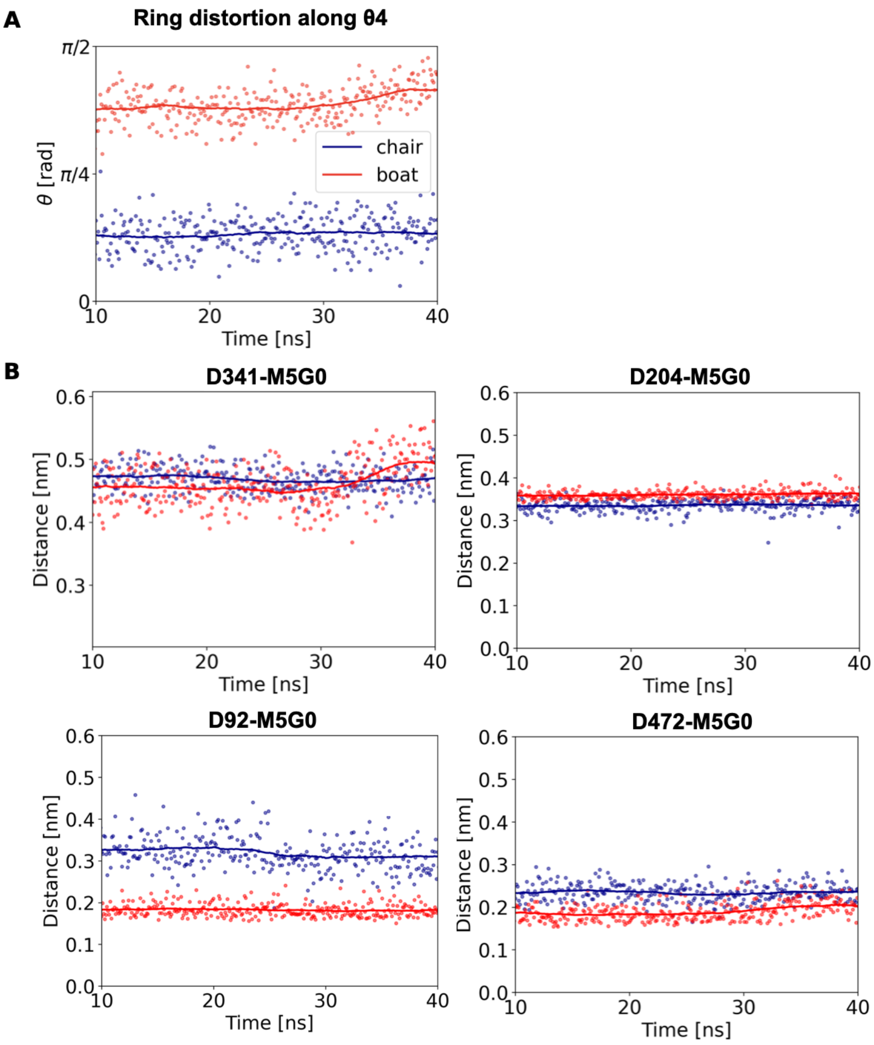

Figure S13: Distances between certain amino acids and the bound glycan M5G0 recorded from **A** two classical MD simulations where Man4 is positioned in the $^{4}C_{1}$ chair (blue) or $^{0}H_{5}$ half-chair (red). The chair was the equilibrium structure derived from the crystal structure. The half-chair was induced by restraining the distances $O_{D92} - H_{O2}$ and $\mathrm{D341_{H}}$ - $\mathrm{M5G0_{O6}}$ to 0.15 nm for 50 ns prior to sampling under unrestraint conditions. **B** Specific distances are $\mathrm{D341_{H}}$ - $\mathrm{M5G0_{O6}}$ (D341-M5G0), $\mathrm{D204_{O1}}$-$\mathrm{M5G0_{HO2}}$ (D204-M5G0), $\mathrm{D92_{O2}}$-$\mathrm{M5G0_{HO2}}$ (D92-M5G0) and $\mathrm{D472_{O1}}$-$\mathrm{M5G0_{HO4}}$ (D472-M5G0). Dots represent individual data points and lines the moving average with a window size of 100 data points.

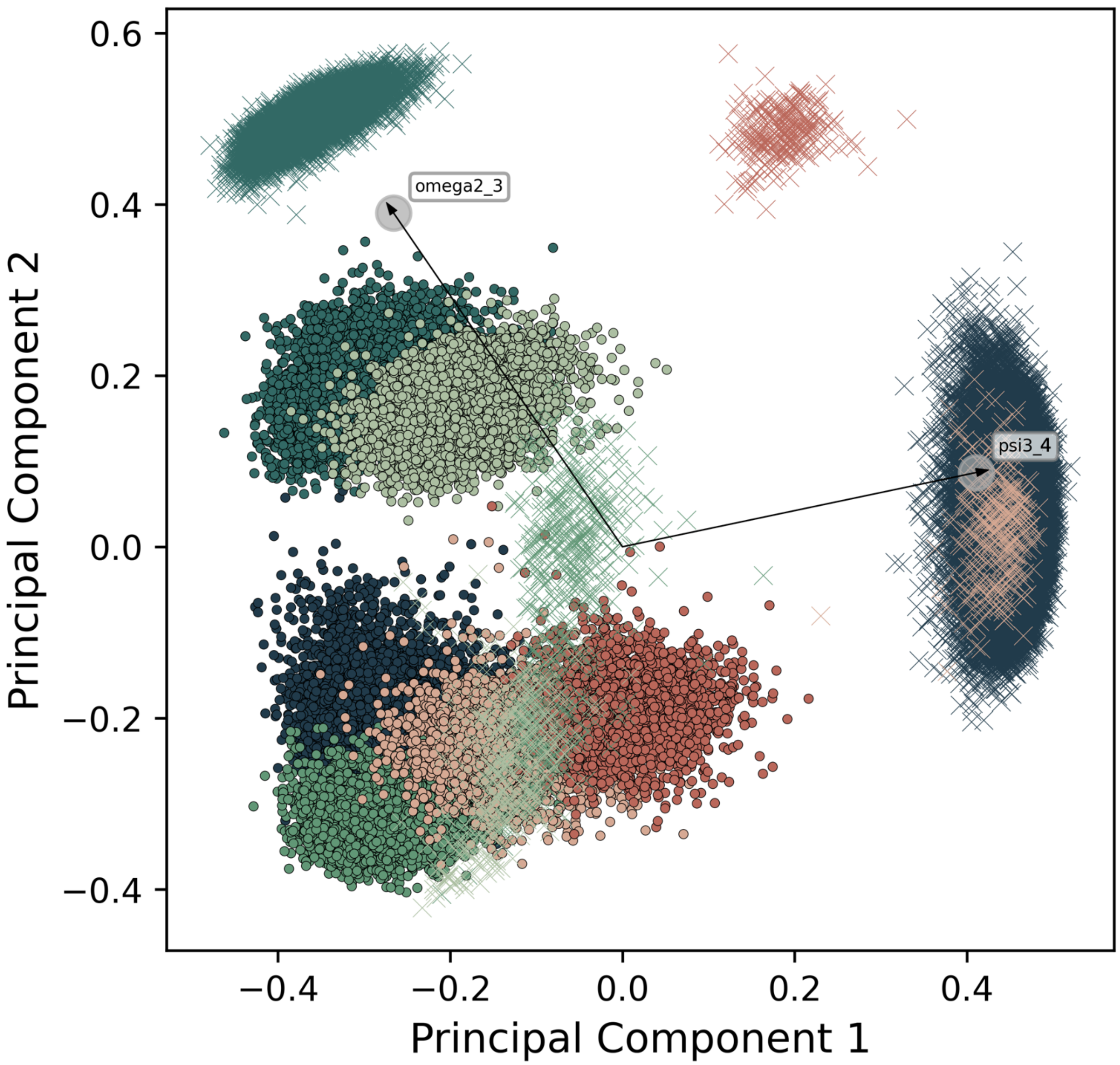

Figure S14: Comparative conformational phase space for M5G0 in solution (dots) and bound to MII (crosses) projected along PC1 and PC2. Only data points corresponding to the 6 most prominent conformers are plotted. Vectors indicate the original feature axes with highest variance, where they point in the direction with highest squared multiple correlation with the principle components. Colors correspond to the conformers and match the once in Figure S4 and S5.

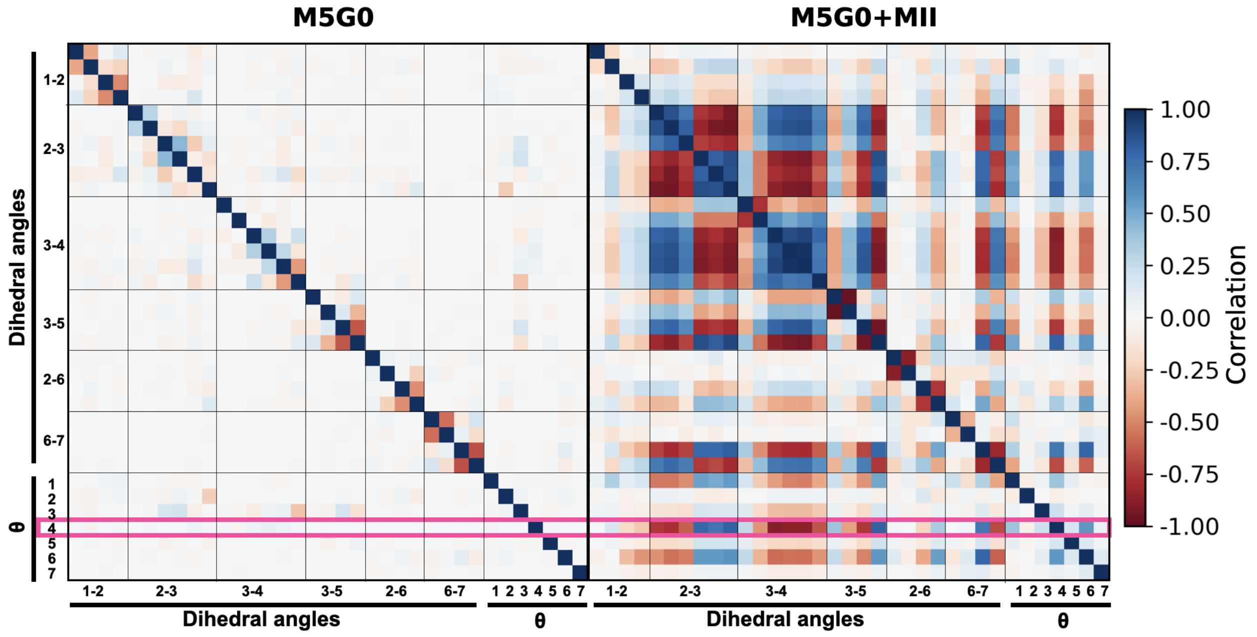

Figure S15: Correlation matrices for M5G0 in solution (left) and bound to MII (right), displaying the Pearson correlation coefficient for all dihedral angles (sin and cos) and θ of all monosaccharides. The pink square highlights the ring distortion of Man4.

Table S1: **Dihedral angle values in rad for conformers of glycan M5G0 sampled in solution (#1) and when bound to MII (#1*, #2*).** Letters in brackets represent the corresponding IUPAC nomenclature letter that is also used in the GlyCONFORMER string, classifying and labeling the respective energy minima of a dihedral angle.

Linkage	Dihedral angles	#1	#1*	#2*
Man2-Man3	φ	+1.3 (G_+)	+1.2 (G_+)	+2.7 (T)
	ψ	-3.0 (T)	-3.1 (T)	-1.9 (A_-)
	ω	-1.2 (gg)	-2.9 (tg)	+1.0 (gt)
Man3-Man4	ψ	-3.0 (T)	+1.1 (G_+)	+3.1 (T)
	ω	-1.2 (gg)	+0.8 (gt)	-1.2 (gg)

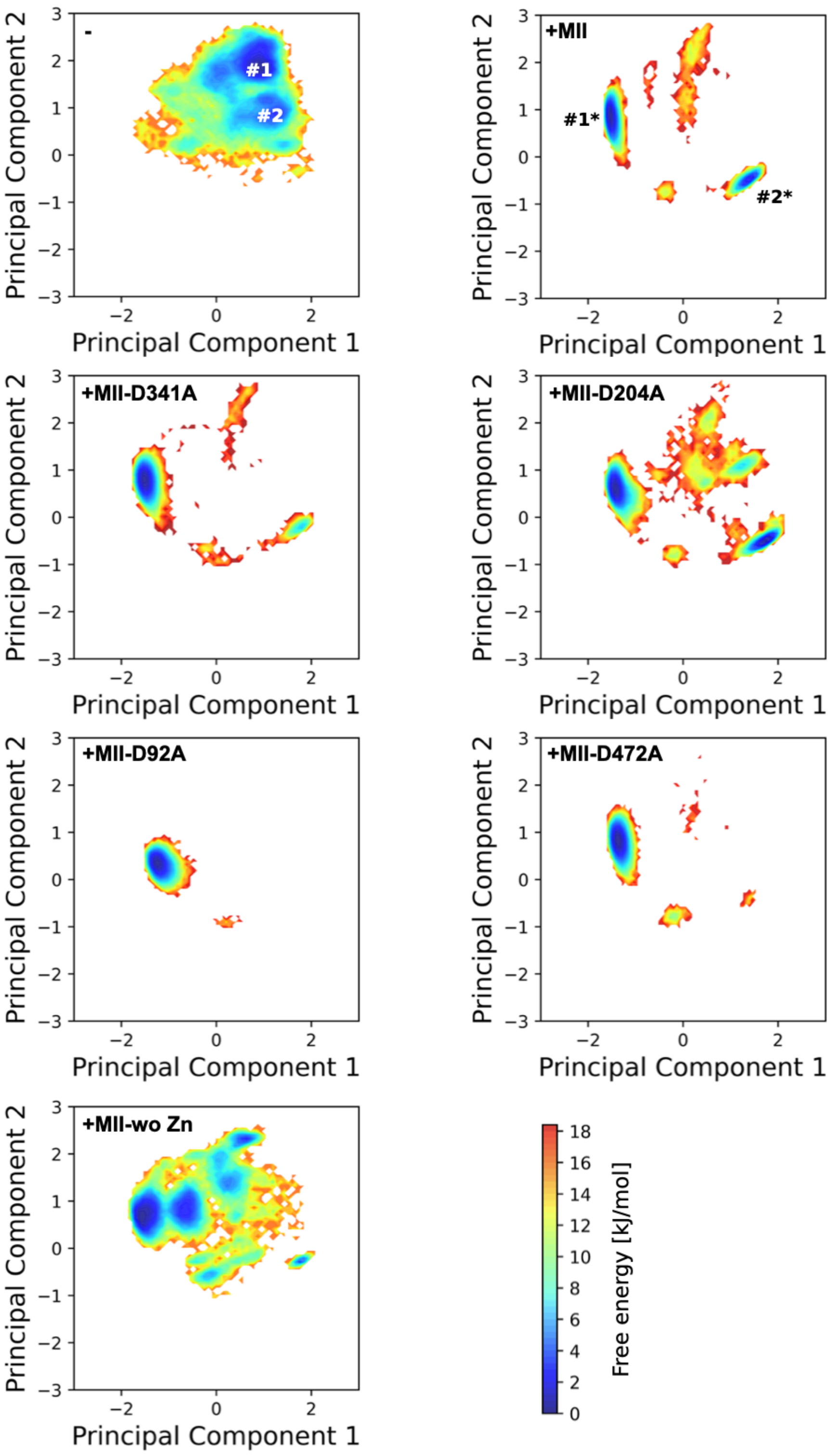

Figure S16: PCA of the comparative conformational phase space for M5G0, M5G0+MII or a mutated variant. First two glycan conformers for M5G0 and M5G0+MII are indicated with labels.

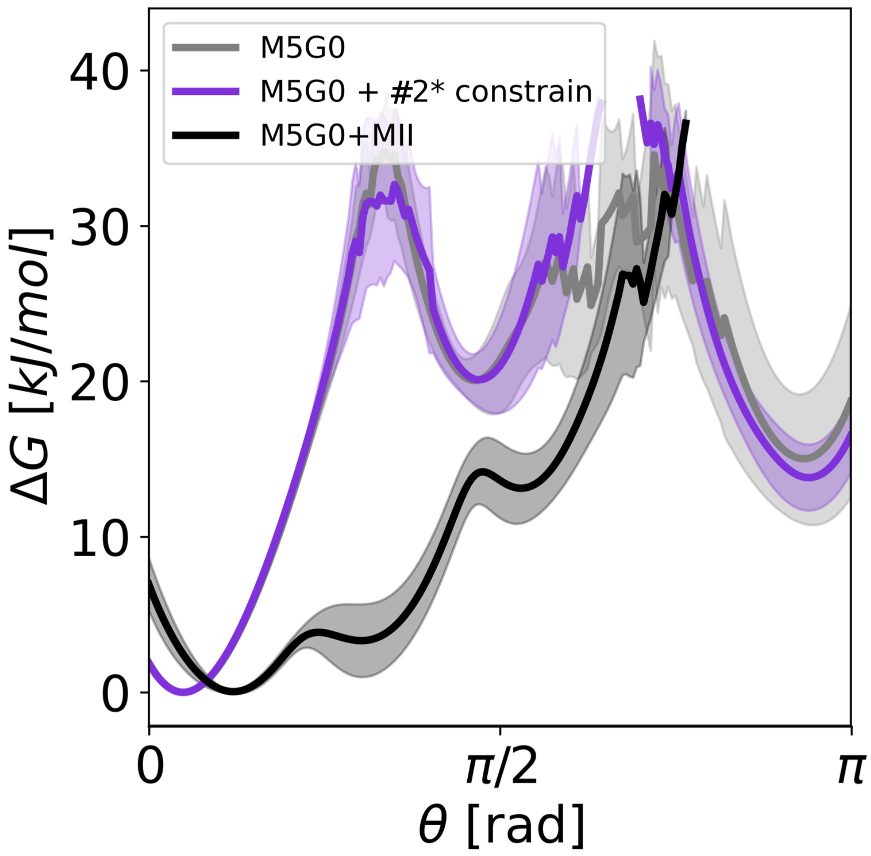

Figure S17: Ring distortion of the terminal mannose residue in glycan M5G0 monitored by the 1D Cremer-Pople parameter θ for M5G0 in aqueous solution (- enzyme), MII's catalytic site at subsite *-1* (+ enzyme) and the conformation M5G0 restricted to the #2* conformer, corresponding structurally to the enzyme bound state that has a distorted ring.

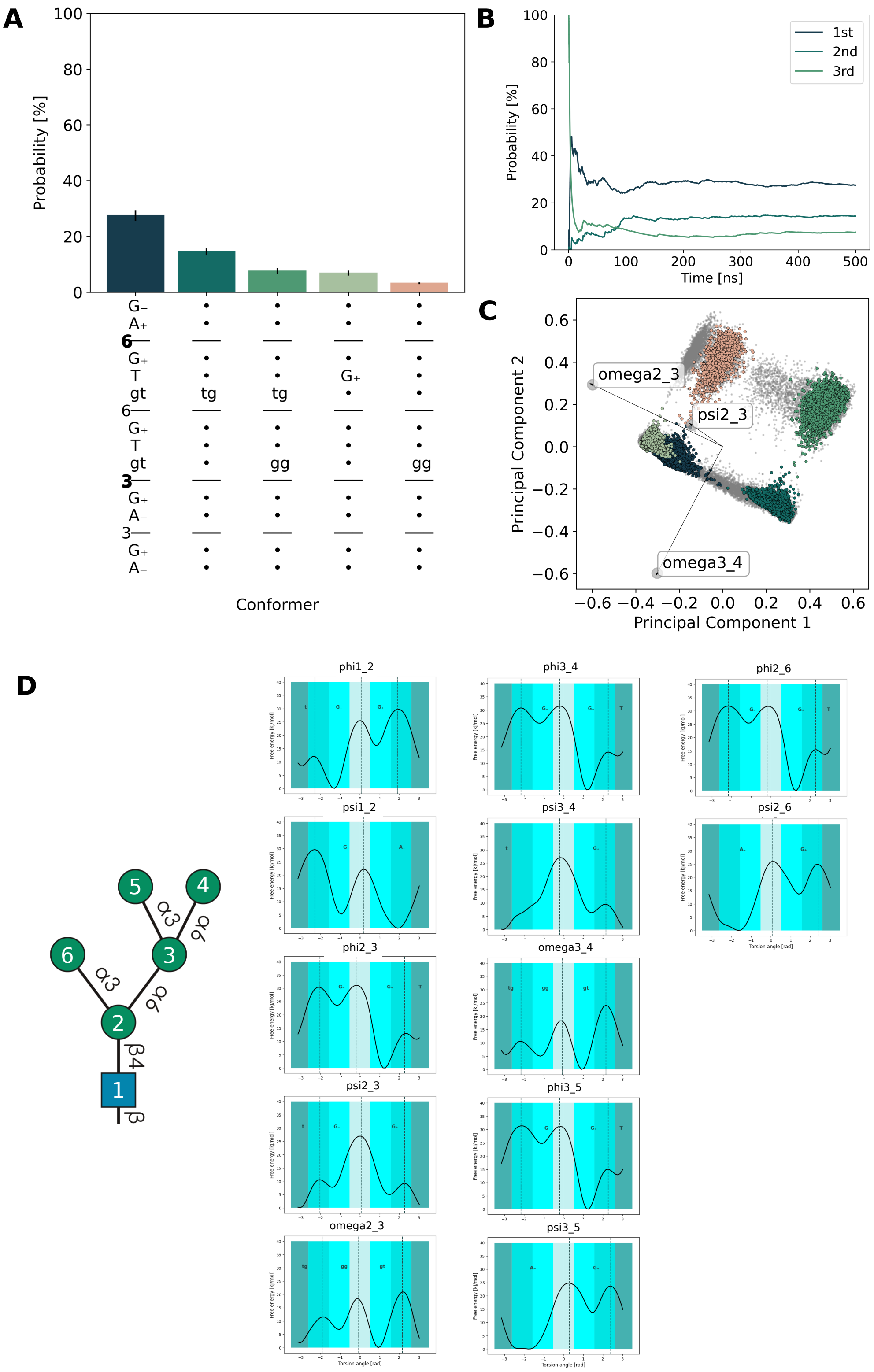

Figure S18: GlyCONFORMER analysis of M5 in solution with same panels as in Figure S4.

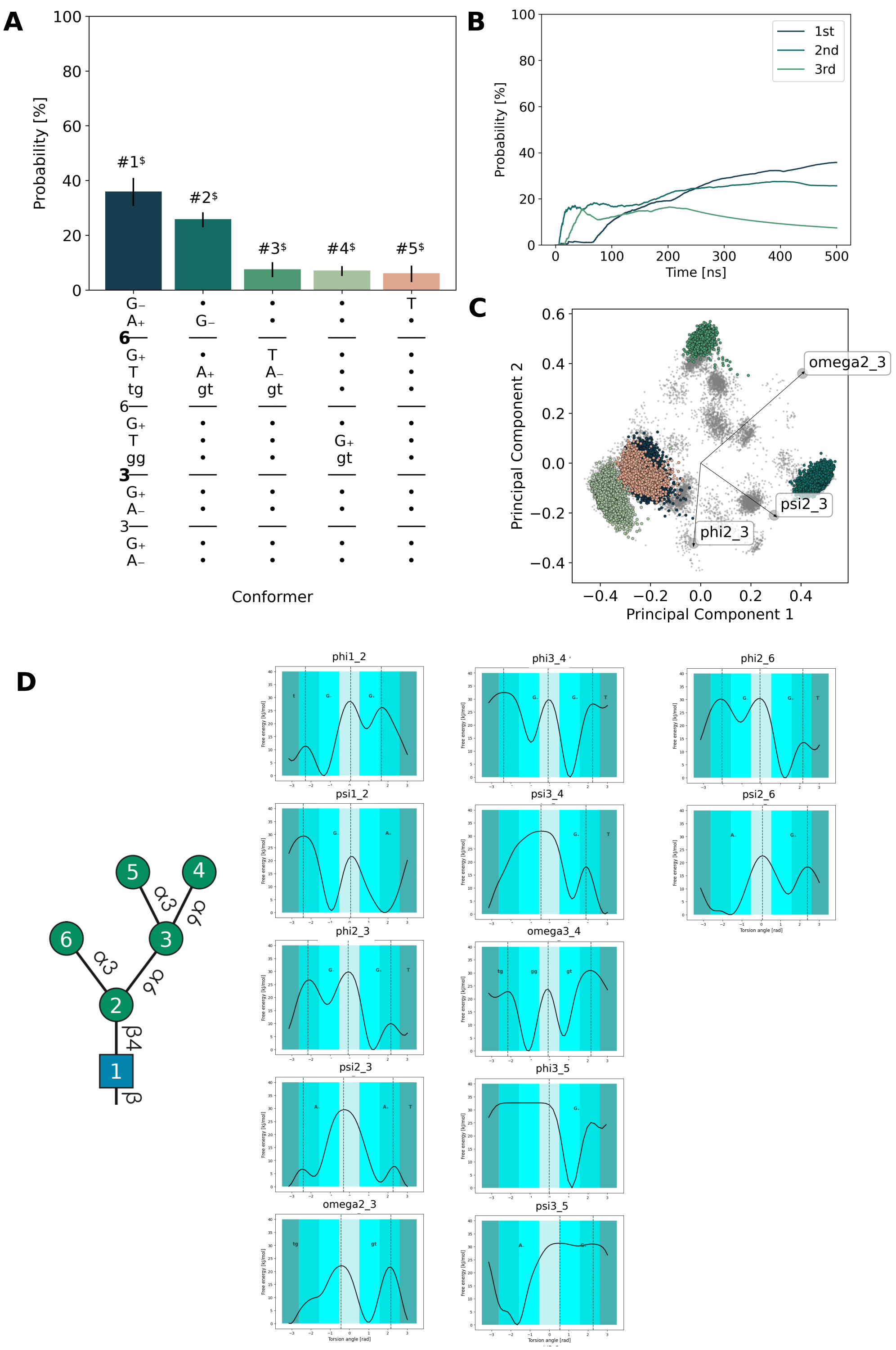

Figure S19: GlyCONFORMER analysis of M5 bound to MII with same panels as in Figure S4.

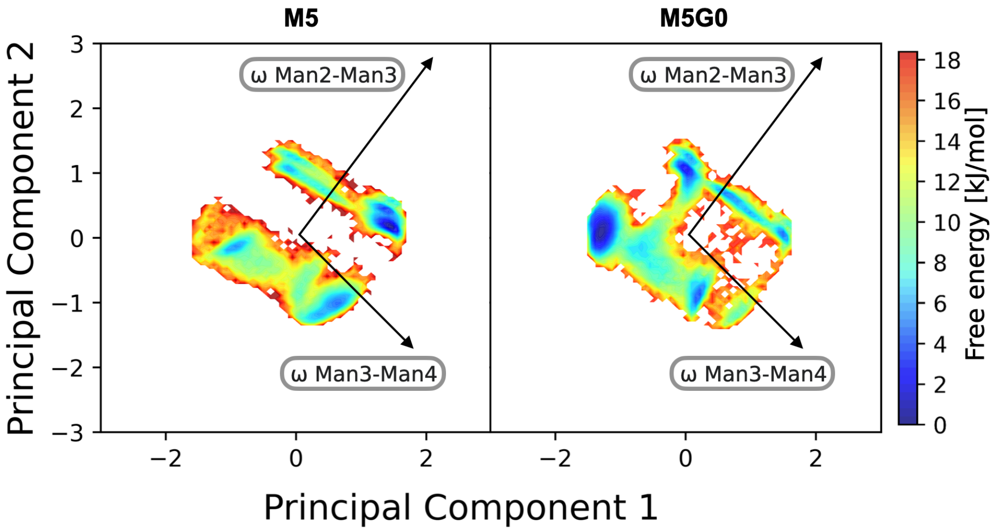

Figure S20: Comparative free energy surfaces of the conformational phase space for M5 and M5G0 projected along PC1 and PC2, with vectors indicating the original feature axes with highest variance.

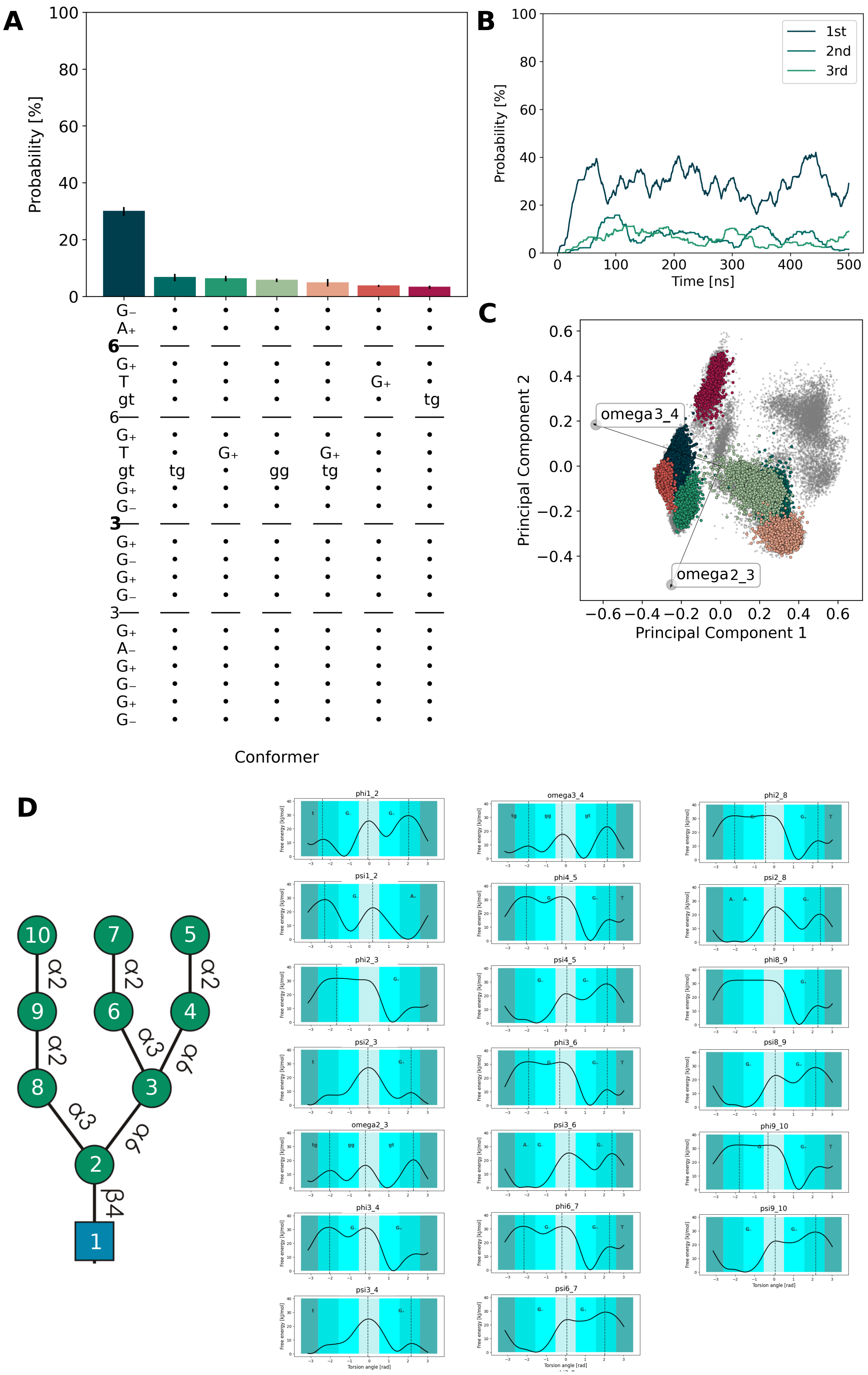

Figure S21: GlyCONFORMER analysis of M9 in solution with same panels as in Figure S4.

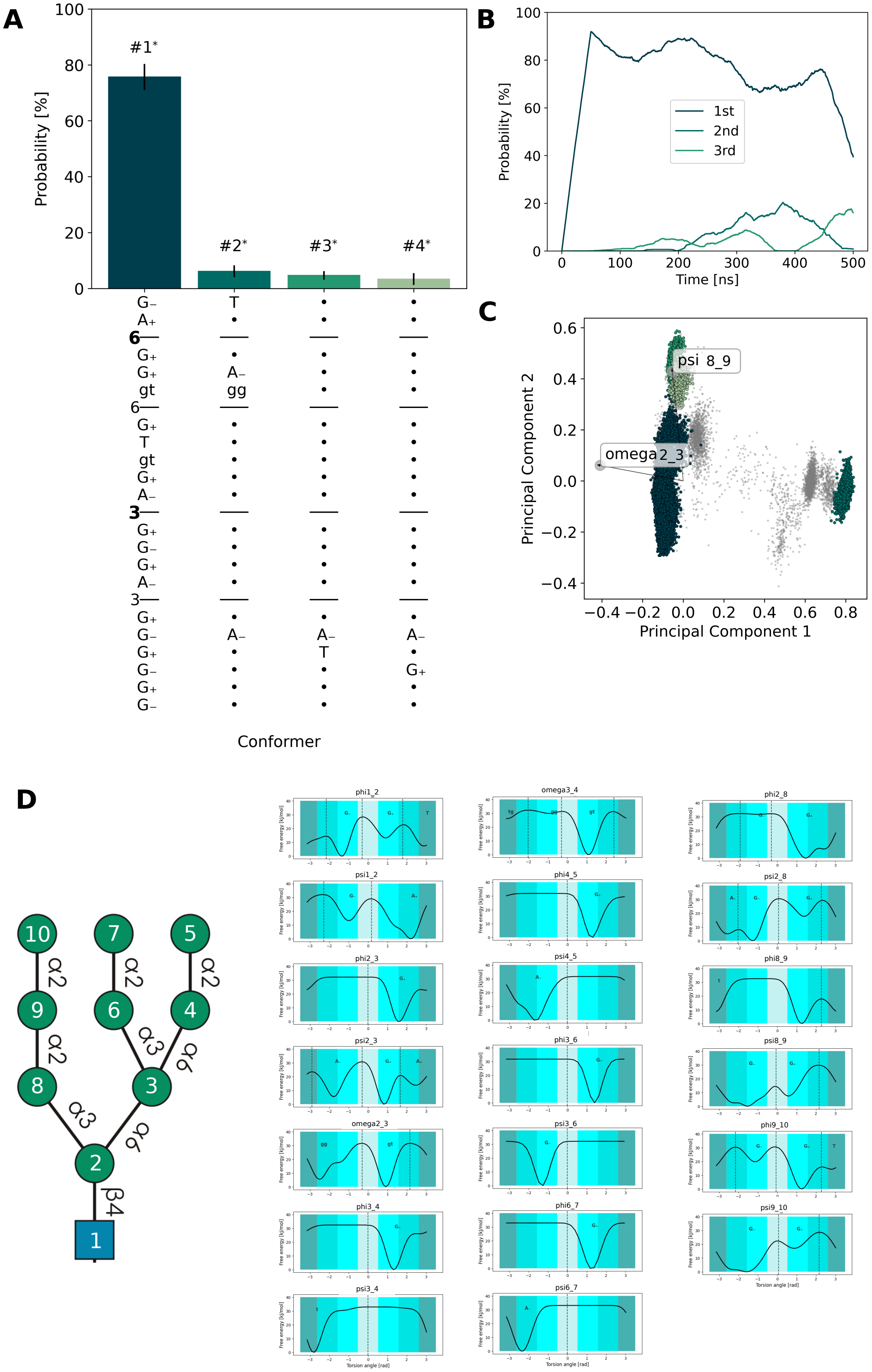

Figure S22: GlyCONFORMER analysis of M9 bound to MI (M9+MI) with same panels as in Figure S4.

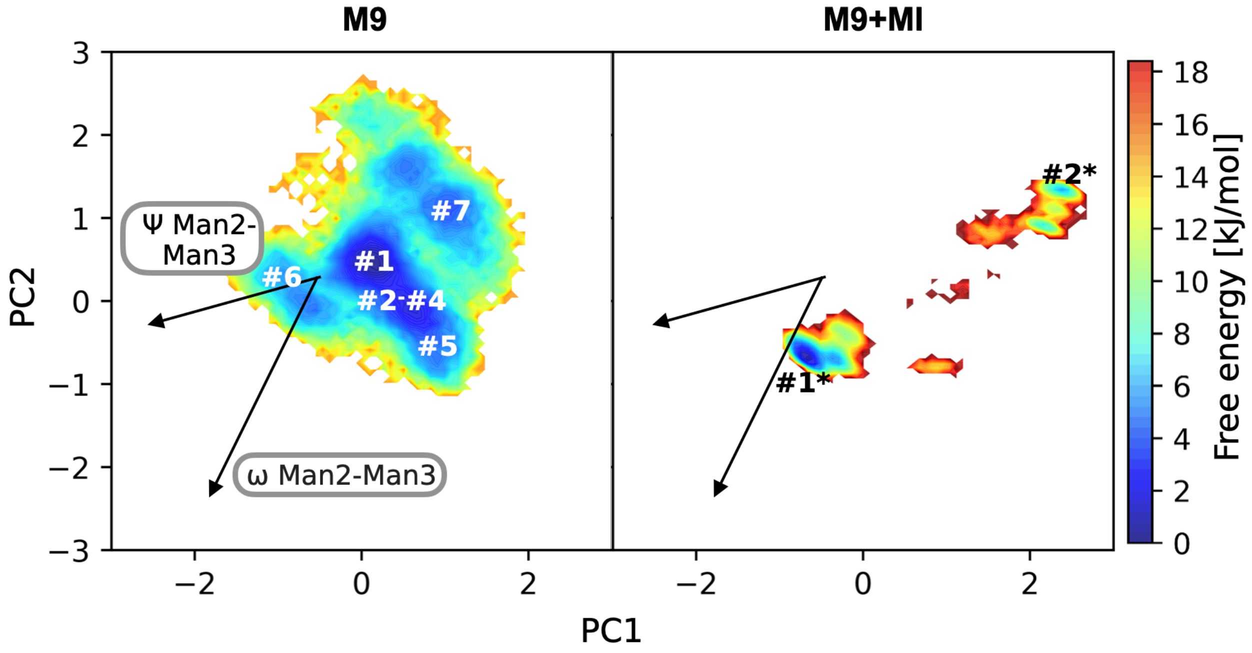

Figure S23: Comparative free energy surfaces of the conformational phase space for M9 and M9+MI projected along PC1 and PC2, with labeled conformers and vectors indicate the original feature axes with highest variance.

References

(S1) Grothaus, I. L.; Bussi, G.; Ciacchi, L. C. Exploration, Representation, and Rationalization of the Conformational Phase Space of N-Glycans. *Journal of Chemical Information and Modeling* **2022**, *62*, 4992–5008.
